# Tunneling nanotubes provide a route for SARS-CoV-2 spreading

**DOI:** 10.1126/sciadv.abo0171

**Published:** 2022-07-20

**Authors:** Anna Pepe, Stefano Pietropaoli, Matthijn Vos, Giovanna Barba-Spaeth, Chiara Zurzolo

**Affiliations:** ^1^ Unité de Trafic Membranaire et Pathogénèse, Département de Biologie Cellulaire et Infection, Institut Pasteur, CNRS UMR3691, Université Paris Cité, 75015 Paris, France.; ^2^ Unité de Virologie Structurale, CNRS UMR 3569 Département de Virologie, Institut Pasteur 28 rue du Docteur Roux, Université Paris Cité, 75015 Paris, France.; ^3^ Catalent Pharma Solutions, Strada Provinciale 12 Casilina, 41, 03012 Anagni, Frosinone, Italy.; ^4^ Plateforme Technologique Nanoimagerie Institut Pasteur, 25 rue du Docteur Roux, 75015 Paris, France.

## Abstract

Neurological manifestations of severe acute respiratory syndrome coronavirus 2 (SARS-CoV-2) infection represent a major issue in long coronavirus disease. How SARS-CoV-2 gains access to the brain and how infection leads to neurological symptoms are not clear because the principal means of viral entry by endocytosis, the angiotensin-converting enzyme 2 receptor, are barely detectable in the brain. We report that human neuronal cells, nonpermissive to infection through the endocytic pathway, can be infected when cocultured with permissive infected epithelial cells. SARS-CoV-2 induces the formation of tunneling nanotubes (TNTs) and exploits this route to spread to uninfected cells. In cellulo correlative fluorescence and cryo–electron tomography reveal that SARS-CoV-2 is associated with TNTs between permissive cells. Furthermore, multiple vesicular structures such as double-membrane vesicles, sites of viral replication, are observed inside TNTs between permissive and nonpermissive cells. Our data highlight a previously unknown mechanism of SARS-CoV-2 spreading, likely used as a route to invade nonpermissive cells and potentiate infection in permissive cells.

## Teaser

Human neuronal cells can be infected by receiving SARS-CoV-2 virus through tunneling nanotubes from preinfected epithelial cells.

## INTRODUCTION

Coronavirus disease 2019 (COVID-19), the disease caused by the severe acute respiratory syndrome coronavirus 2 (SARS-CoV-2), has been developing into a global pandemic since the first reported events in December 2019 (*1*, *2*). Although SARS-CoV-2 primarily targets the respiratory tract and most patients with COVID-19 present severe respiratory symptoms (*3*), other organs such as the intestine, liver, kidneys, heart, and brain can also be affected. Neurological manifestations of different gravity have also been reported (*4*–*7*). The neurological symptoms can be acute and resolve with the disease or can represent a major issue in the case of long COVID (*8*–*10*). The ability of SARS-CoV-2 to enter the central nervous system (CNS) is expected given that several types of CoV have been reported to invade and persist in the CNS (e.g., SARS-CoV and Middle East respiratory syndrome–CoV) (*11*, *12*). In addition, case reports have shown that the brain tissue of patients that died following COVID-19 were positive for SARS-CoV-2 RNA (*13*).

Investigating how SARS-CoV-2 enters neuronal cells is essential for understanding the neurological manifestations associated with COVID-19. However, how SARS-CoV-2 gains access to the CNS and how infection leads to neurological symptoms are still not clear (*14*–*18*). SARS-CoV-2 neuroinvasion could be achieved through several routes (*19*), and once it reaches the CNS, it could bind the angiotensin-converting enzyme 2 (ACE2) receptor exposed on neuronal cells to infect the brain (*20*). The ACE2 receptor is the main actor responsible for virus entry in the lower respiratory tract (*13*, *21*, *22*). To enter host cells, the viral spike (S) proteins of CoVs bind the enzymatic domain of the ACE2 receptor. To gain access to the cytosol, SARS-CoV-2 must fuse its envelope with the cell membranes. This is mediated by the proteolytic activation of the S protein that can occur at the endosomes following endocytosis, whereby endosomal acidification triggers endolysosomal proteases priming viral fusion (*23*). Alternatively, in the presence of TMPRSS2 (transmembrane serine protease 2) (*24*) at the plasma membrane (PM), after binding to ACE2 receptor, SARS-CoV-2 uses a fast pH-independent route to enter cells, which allows direct fusion of the virus with the PM (*23*). The ACE2 receptor is exposed on the surface of the cells forming the oral cavity and the oropharynx (*24*–*26*). While the expression of the ACE2 receptor has been well documented in many cell types and tissues (*24*, *25*), in the human brain, ACE2 receptor levels are very low, with the exception of brain areas such as the thalamus and the choroid plexus (*27*). For this reason, it is not clear how the virus can propagate through the brain. Here, we investigated the neuroinvasive potential of SARS-CoV-2 and asked whether tunneling nanotubes (TNTs) could be involved in its intercellular spreading. TNTs are thin, membranous conduits rich in actin (*28*, *29*) that allow the direct transport of cargos including organelles, amyloid proteins (*28*, *30*), and viral particles between distant cells (*30*–*35*). We hypothesized that SARS-CoV-2 could use TNTs to spread from permissive cells to less-permissive cells that lack the membrane receptor for virus entry, thus allowing the spreading of viral pathogenicity and escaping from immune surveillance. To test this hypothesis, we used the Vero E6 cell line as an epithelial model because it has been widely used for SARS-CoV-2 isolation, propagation, and antiviral testing (*36*–*38*). As a neuronal model of nonpermissive cells, we used the SH-SY5Y cell line; these are human cells widely used as a neuronal model, and their TNTs have also been thoroughly characterized (*39*) and are identifiable with high reliability (*39*, *40*). While primary neurons would have been preferable, it is unfortunately very difficult to discriminate TNT-like structures (*41*), and it is even more challenging to apply the advanced cryo-correlative light and electron microscopy (CLEM) and cryo-electron tomography (ET) approaches that we have developed here.

By using confocal microscopy and establishing in cellulo cryo-CLEM and cryo-ET (*39*), we demonstrate that SH-SY5Y human neuronal cells, not permissive to SARS-CoV-2, can be infected through a TNT-mediated mechanism when cocultured with infected Vero E6–permissive epithelial cells. Together, our results reveal the structure of the viral particles associated with TNTs and provide information about the molecular mechanism of SARS-CoV-2 infection and transmission. Within the limitations of an in vitro study, these data support the role of TNTs in viral spreading, possibly enhancing the efficiency of viral propagation through the body.

## RESULTS

### SARS-CoV-2 can spread among cells independently of receptor-mediated endocytosis

**Fig. 1.**
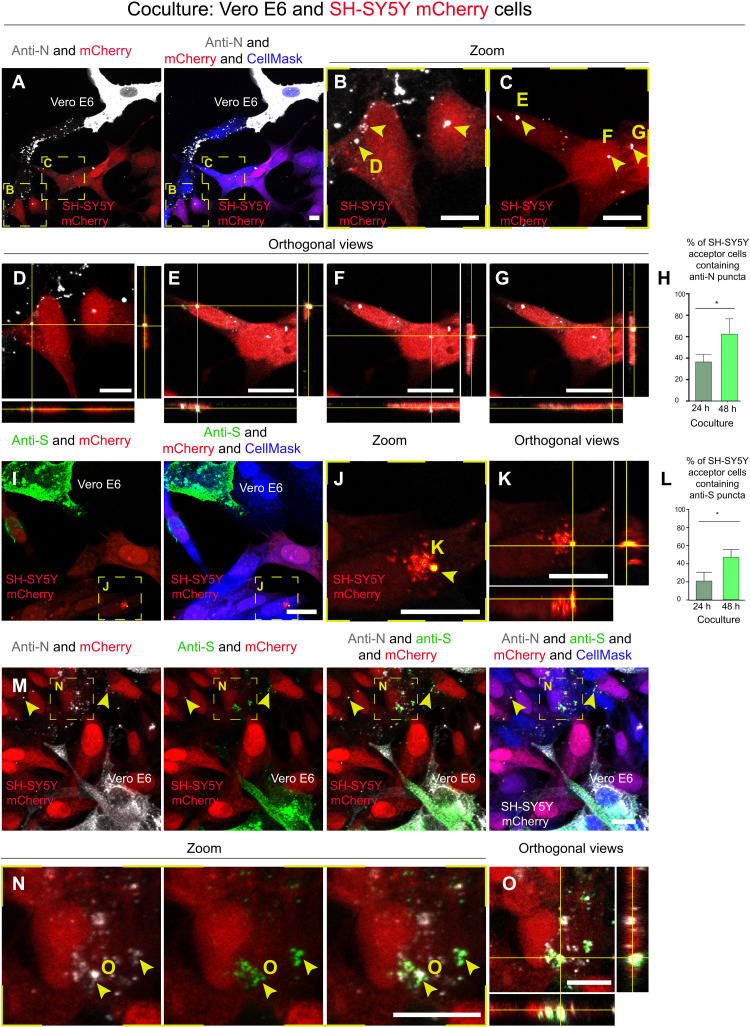
SARS-CoV-2 can reach SH-SY5Y neuronal cells from Vero E6 permissive cells. Infected Vero E6 cells (donor cells) were cocultured with SH-SY5Y neuronal cells previously stably transfected with a vector that expresses mCherry (acceptor cells). Coculture was fixed at 24 and 48 hours. (**A** to **G**) Confocal micrographs showing 48 hours of coculture between SARS-CoV-2–infected Vero E6 cells and SH-SY5Y mCherry cells. An anti-N antibody was used to detect SARS-CoV-2 nucleoproteins. (B and C) Enlargement of the yellow dashed squares in (A); the yellow arrowheads indicate the anti-N puncta detected in the cytoplasm of acceptor cells. (D to G) The orthogonal views of (B) and (C) showing the anti-N puncta inside the cytoplasm of acceptor cells. (**H**) Graph showing the mean percentage of anti-N puncta transferred to acceptor cells after 24 and 48 hours of coculture. *P = 0.0468. (**I** to **K**) Confocal micrographs showing 48 hours of coculture between SARS-CoV-2–infected Vero E6 cells and SH-SY5Y mCherry cells. An anti-S antibody was used to detect SARS-CoV-2 particles. (J) Enlargement of the yellow dashed square in (I); the yellow arrowhead indicates the anti-S puncta in the acceptor cells. (K) The orthogonal views of (J) showing the anti-S puncta inside acceptor cells. (**L**) Graph showing the mean percentage of anti-S puncta transferred to acceptor cells after 24 and 48 hours of coculture. *P = 0.0374. (**M** to **O**) Double immunostaining of coculture using anti-S and anti-N antibodies. (N) Enlargement of the yellow dashed square in (M) showing colocalization between anti-N and anti-S puncta in SH-SY5Y mCherry acceptor cells. The cytosol has been labeled with CellMask Blue. Scale bars, 10 μm.

We first tested different neuronal cells to verify their permissiveness to viral infection by a receptor-mediated endocytic pathway. Human (SH-SY5Y) and murine [Cath.a-differentiated (CAD)] neuronal cell lines were infected with a range of MOI (multiplicity of infection). After 3 days, we looked for productive infection by staining the infected monolayers with anti–nucleoprotein (N) virus-specific antibody and by titrating the virus released in the supernatant (fig. S1, A and B). Neither cell line showed any sign of infection or viral production (fig. S1, A and B). By contrast, control epithelial Vero E6 and Caco-2 cells were susceptible to infection with SARS-CoV-2, as previously shown (fig. S1, A and B) (*36*). These data show that neuronal cells cannot be infected directly from the supernatant through a receptor-mediated mechanism. Consistently, we were unable to detect a signal for ACE2 in SH-SY5Y cells (fig. S2), confirming previous observations reporting extremely low levels of expression in neuronal cells (*42*). An alternative possibility is that the virus exploits intercellular communication pathways to enter neuronal cells directly from permissive cells. To investigate this, we set up coculture experiments between permissive Vero E6 cells and nonpermissive SH-SY5Y human neuronal cells. Vero E6 cells (donor cells) infected with SARS-CoV-2 at an MOI of 0.05 for 48 hours were cocultured with SH-SY5Y cells (acceptor cells) previously transfected with a plasmid encoding mCherry to distinguish them from donor cells (fig. S3A). After 24 and 48 hours of coculture, cells were fixed and immunostained with anti-N antibody, recognizing SARS-CoV-2 N and labeled with CellMask Blue to stain the whole cells (Fig. 1, A to G). By using confocal microscopy and Icy software (icy.bioimageanalysis.org), we calculated the percentage of SH-SY5Y acceptor cells positive for the anti-N antibody immunostaining. After 24 hours of coculture, 36.4% of acceptor cells contained spots recognized by the anti-N antibody in their cytoplasm (Fig. 1H), and this percentage increased to 62.5% after 48 hours (Fig. 1, A to H).

To further investigate the nature of the viral particles present in acceptor neuronal cells, additional cocultures between infected Vero E6 cells and SH-SY5Y neuronal cells were immunostained (after 24 and 48 hours of coculture) using an anti-S antibody, both alone (Fig. 1, I to K) and in combination with the anti-N antibody (Fig. 1, M to O). Similar to the results obtained with the N antibody, we found that after 24 hours of coculture, 21.8% of acceptor cells contained spots positive for the anti-S antibody, and after 48 hours of coculture, this value increased to 42.4% (Fig. 1L). We then evaluated the colocalization of the two viral proteins N and S in the acceptor cells (Fig. 1, M to O) and found that at 48 hours, the Pearson’s correlation coefficient (PCC) was, on average, 0.716, indicating that the two proteins partially colocalize. At 24 hours, we found negligible colocalization between the two proteins, so we could not calculate the PCC; therefore, we believe that there is an evolution of the infection over time. While separate signals for anti-N and anti-S antibodies could be suggestive of virus uncoating during the first step of the infection (*43*), colocalization of N and S proteins in nonpermissive SH-SY5Y cells could correspond to mature virions inside endocytic vesicles that are transferred directly from infected Vero E6 cells and/or to newly synthesized virions assembled in the neuronal acceptor cells.

**Fig. 2.**
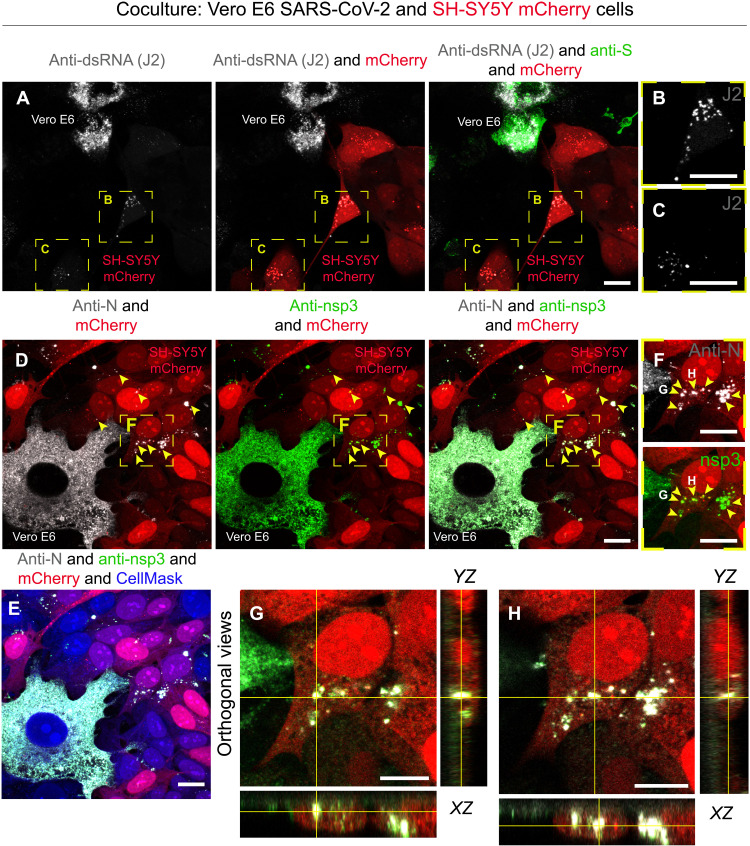
Anti-dsRNA antibody J2 and the nonstructural protein 3 are detected in SH-SY5Y cells cocultured with SARS-CoV-2–infected Vero E6 cells. (**A**) SARS-CoV-2–infected Vero E6 cells (donor cells) were cocultured for 48 hours with SH-SY5Y mCherry acceptor cells. Confocal micrographs showing the staining with J2 antibody are used to detect dsRNA, and an anti-S antibody is used to detect SARS-CoV-2 particles. (**B** and **C**) Enlargement of the yellow dashed squares in (A). (**D** to **H**) Confocal micrographs showing 48 hours of coculture of SARS-CoV-2–infected Vero E6 cells (donor) and SH-SY5Y mCherry cells (acceptor) stained using anti–nonstructural protein 3 (nsp3) and anti-N antibodies. (E) Confocal micrographs showing cellular cytoplasm labeled with CellMask Blue. (F) Enlargement of the yellow dashed square in (D) showing puncta positive for both anti-nsp3 and anti-N in acceptor cells. (G and H) Confocal micrographs representing the orthogonal views of (F) showing anti-nsp3 and anti-N puncta in the cytoplasm of acceptor cells. Yellow arrowheads indicate anti-nsp3 and anti-N signal in acceptor cells. Scale bars, 10 μm.

To directly investigate whether SARS-CoV-2 was able to replicate in neuronal cells, we performed an immunostaining using the anti–dsRNA (double-stranded RNA) antibody J2, the current gold standard for the detection of dsRNA in infected cells (*44*). After 48 hours of coculture (infected Vero E6 cells as donors and SH-SY5Y cells as acceptors), cells were fixed and immunostained for dsRNA. We found J2-positive signal both in donor infected Vero E6 cells and in acceptor SH-SY5Y cells (Fig. 2, A to C). Notably, the J2 signal in SH-SY5Y cells corresponds to bright foci (Fig. 2, B and C); these could represent replication organelles or double-membrane vesicles (DMVs), where viral RNA synthesis occurs (*45*, *46*). Furthermore, the localization of J2 spots in the perinuclear/endoplasmic reticulum region of acceptor cells could suggest active viral replication in the neuronal cells (Fig. 2, B and C) (*47*–*51*). Accordingly, the J2 signal was specific for infected cells in coculture, as it was not detected in noninfected Vero E6 cells cocultured with SH-SY5Y mCherry cells (fig. S3B) and neither in the negative control in which the coculture was incubated only with the secondary antibody (fig. S3C).

As a complementary approach, to support the hypothesis that SARS-CoV-2 transferred to neuronal cell acceptors could actively replicate within them, we performed an immunostaining against the nonstructural protein 3 (nsp3), an essential component of the viral replication/transcription complex (*46*). After 48 hours of coculture (infected Vero E6 cells as donors and SH-SY5Y cells as acceptors), cells were fixed and immunostained to detect nsp3 and N proteins of SARS-CoV-2. We found cytoplasmic puncta positive for both the N and nsp3 proteins in the acceptor SH-SY5Y mCherry cells (Fig. 2, D to H). Consistent with recent studies (*52*), we found colocalization of nsp3 and KDEL in both acceptor and donor cells (fig. S4A), while there was no colocalization between giantin and dsRNA (revealed with J2 antibody) in SH-SY5Y cells and infected Vero E6 cells (fig. S4B). Next, to better understand whether the viral signal found within the SH-SY5Y acceptors cocultured with infected donors was due to de novo replicated virus or virus coming from infected permissive donor, we treated cocultures with the viral replication inhibitor remdesivir. To determine the minimal concentration of the inhibitor, sufficient to block viral replication, we preincubated Vero E6 mCherry cells for 1 hour at 37°C, with three different concentrations of remdesivir (3, 30, and 40 μM) and maintained the inhibitor for the duration of the infection (SARS-CoV-2 at an MOI of 0.05). Viral production was then assessed at 48 hours by immunostaining with the J2 and anti-S antibodies. Both 30 and 40 μM remdesivir were enough to inhibit virus production (fig. S5A) and did not show toxicity; therefore, the lower effective concentration of 30 μM was chosen. Infected Vero E6 donor cells were put in coculture with SH-SY5Y mCherry acceptors treated with the inhibitor. As observed previously (Fig. 1L), after 48 hours under coculture control conditions, around 45% of acceptor SH-SY5Y cells were positive for anti-S and 40% were positive for J2 immunostaining (fig. S5, B to D). This percentage decreased markedly to 15.3% for anti-S and 17.2% for J2 in the coculture, where the acceptors were treated with the remdesivir (fig. S5, B to D), indicating that newly synthesized virions can be assembled in the neuronal acceptor cells. As expected, no infection could be detected in the SH-SY5Y cells (incubated or not with the remdesivir) challenged with the supernatant of infected cells (fig. S5E). As control for the efficiency of the inhibitor blockage of replication, we challenged naïve Vero E6 mCherry cells (incubated and not with the remdesivir) with the supernatants of infected Vero E6. While 100% of control cells were infected (e.g., positive for SARS-CoV-2 J2 and S staining) (fig. S5F), no infection could be detected in the cells treated with the inhibitor (fig. S5F).

### TNTs contribute to SARS-CoV-2 transmission

While the above data show that neuronal cells can be infected when in coculture with permissive cells, they do not address the mechanism. To rule out any possible contribution of virus uptake from the cellular medium under our coculture conditions, we performed “secretion tests” in which supernatants from infected Vero E6 cells were used to infect either SH-SY5Y cells or control Vero E6 cells (cultured separately) for 24 and 48 hours (fig. S6, A to D). As expected from our previous screens (fig. S1), we did not detect any notable signal for either the anti-N or anti-S antibodies in the acceptor cells that received the supernatants from the infected Vero E6 cells (fig. S6B), contrary to Vero E6 cells that resulted positive for both anti-N and anti-S antibodies (fig. S6C). In addition, the 48-hour supernatants from donor-infected, coculture, and secretion experiments were used to assess viral production by focus-forming assay titration protocol (fig. S6D).

**Fig. 3.**
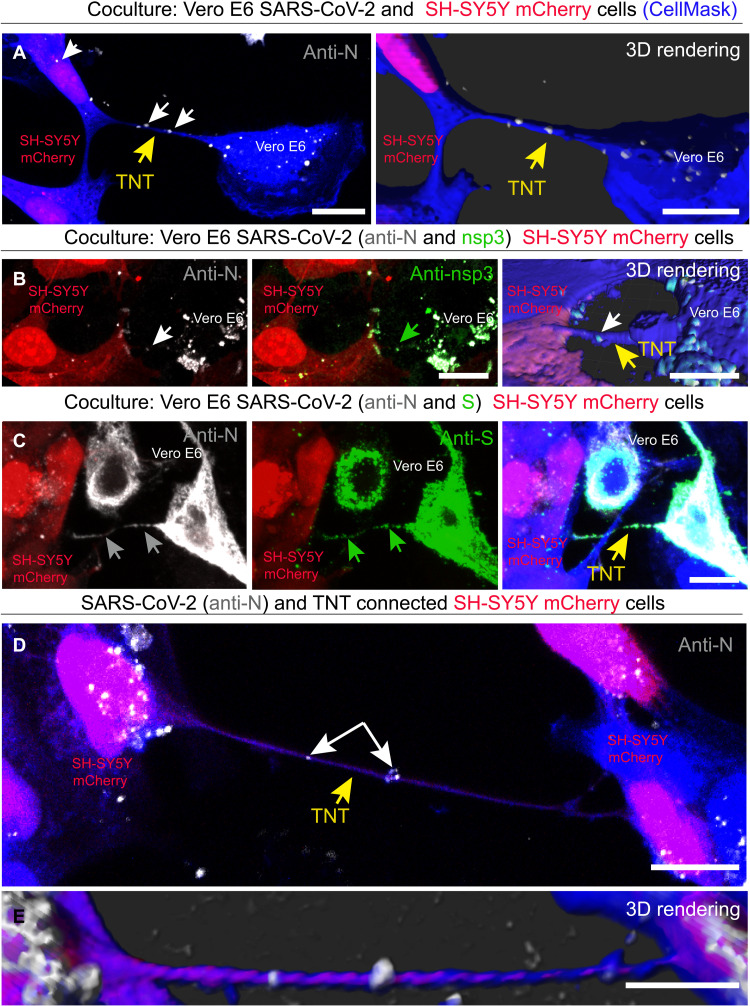
SARS-CoV-2 spread through TNTs from infected Vero E6 to noninfected SH-SY5Y mCherry cells. (**A**) SARS-CoV-2–infected Vero E6 cells were cocultured with SH-SY5Y mCherry cells. Coculture was fixed at 48 hours and stained with the anti-N antibody to detect the virus. Two-dimensional (2D) confocal micrograph (left) and 3D rendering performed by Imaris software (right), showing a TNT connecting SARS-CoV-2–infected Vero E6 cells and SH-SY5Y mCherry cell. The yellow arrows point the TNT between Vero E6 and SH-SY5Y mCherry cells; the white arrows indicate SARS-CoV-2 anti-N signal inside TNT and in the acceptor cells. (**B**) 2D confocal micrograph and 3D rendering show a TNT connecting infected Vero E6 cells and SH-SY5Y mCherry cells. The TNTs connecting Vero E6 and SH-SY5Y mCherry cells are shown in the 3D rendering. Coculture was stained with the anti-N and anti-nsp3 antibodies. The white arrows indicate the SARS-CoV-2 anti-N signal, and the green arrows indicate the anti-nsp3 signal inside TNT. The yellow arrow points the TNT. (**C**) Coculture was stained with the anti-N and anti-S antibodies to detect the virus. The gray arrows indicate the SARS-CoV-2 anti-N signal, and the green arrows indicate the anti-S signal inside TNT. The yellow arrow points the TNT. (**D** and **E**) 2D confocal micrograph (D) and 3D rendering (E) showing a TNT connecting two SH-SY5Y mCherry cells, cocultured with infected Vero E6 cells. The yellow arrow points the TNT between the SH-SY5Y mCherry cells. The white arrows indicate SARS-CoV-2 inside the TNT. Cellular cytoplasm and TNTs were labeled with CellMask Blue. Scale bars, 10 μm.

Overall, the results described above provide evidence that while SARS-CoV-2 entry into epithelial cells is mediated by the classical receptor-mediated endocytosis pathway, the spreading between permissive cells and nonpermissive neuronal cells could occur through a direct cell-to-cell contact–dependent pathway. We therefore explored whether TNTs could mediate the spreading of SARS-CoV-2 to nonpermissive neuronal cells. To properly identify TNTs by confocal microscopy, it is crucial to distinguish them from other actin-based membranous protrusions such as filopodia (*53*, *54*). TNTs hover above the substrate and even over other cells, and unlike dorsal filopodia, they directly connect two or more distant cells (*39*). On the basis of these criteria, when we cocultured SH-SY5Y cells with infected Vero E6 cells, we observed TNTs between SARS-CoV-2–infected donor cells and SH-SY5Y mCherry acceptor cells containing particles stained with anti-N antibody, which were also found inside the cytoplasm of the neuronal cells (Fig. 3A). Specific labeling using anti-nsp3 and anti-S antibodies revealed that N colocalized with both S and nsp3 proteins (Fig. 3, B and C), suggesting that both replicative complexes and mature virions could be found in TNTs formed between permissive and nonpermissive cells. We also found TNTs between SH-SY5Y cells, which contained anti-N–labeled particles (Fig. 3, D and E). Together, the data presented above (Figs. 1 to 3) indicate that SARS-CoV-2 infection can be transferred in a cell-to-cell contact–dependent manner (likely TNT-mediated) from permissive cells to nonpermissive neuronal cells. Following infection, TNTs appear to be longer (with an average length of 34.48 μm; SD = 24.87; min = 11.87 μm, max = 109.45 μm) compared to control conditions (with an average length of 7.98 μm; SD = 3.65) (fig. S7, A and B). Furthermore, TNT counting under both control and infected conditions revealed that the percentage of TNTs connecting Vero E6 and SH-SY5Y cells increased from 35% under control conditions to 61.75% following SARS-CoV-2 infection (fig. S7C), suggesting that SARS-CoV-2 infection promotes and/or stabilizes TNT formation. Last, the evidence showing N-labeled particles in TNTs between neuronal cells suggests a mechanism whereby SARS-CoV-2 can further propagate from neuron to neuron.

### TNTs facilitate SARS-CoV-2 transmission between permissive cells

**Fig. 4.**
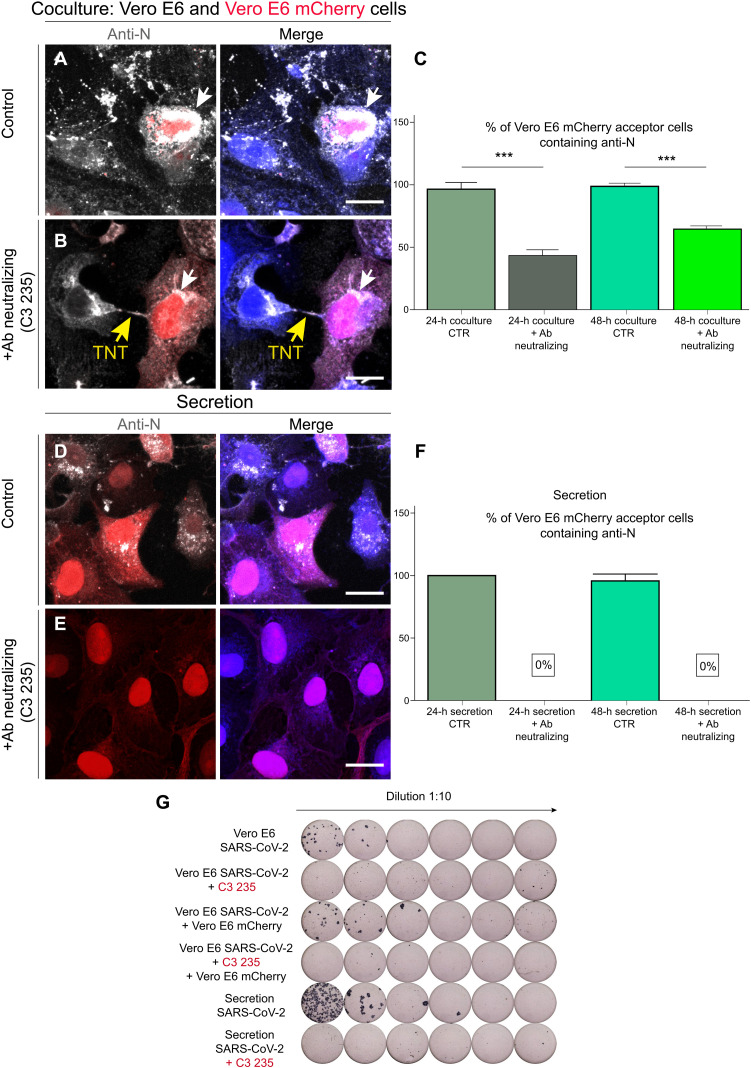
SARS-CoV-2 viral particles spread between permissive cells through TNTs. (**A**) Donor infected Vero E6 cells were put in coculture at 1:1 ratio with Vero E6 mCherry acceptors under control conditions (without neutralizing antibody) and (B) under neutralizing conditions. (**B**) Donor infected Vero E6 cells were incubated with the anti–SARS-CoV-2 IgG C3 235 before being cocultured with Vero E6 mCherry acceptors cells. The cocultures were fixed after 48 hours of incubation and immunostained with anti-N antibody (Ab) to detect SARS-CoV-2. Cellular cytoplasm was labeled with CellMask Blue. (**C**) Graph showing the mean percentage of anti-N puncta transferred in coculture at 24 and 48 hours, treated and not with the neutralizing antibody. The white arrows indicate SARS-CoV-2 anti-N signal, and the yellow arrows point to the TNT. CTR, control. (**D**) Vero E6 mCherry cells were incubated with the supernatant deriving from donor infected Vero E6 cells. (**E**) The supernatant from donor infected Vero E6 cells was incubated with the anti–SARS-CoV-2 IgG C3 235, to neutralize the viral particles, before being added on top of Vero E6 mCherry acceptor cells. After 48 hours of incubation, the secretion samples were fixed and immunostained for anti-N. (**F**) Graph showing the mean percentage of anti-N puncta contained in acceptor cells in the secretion experiments at 24 and 48 hours, treated or not with the neutralizing antibody. (**G**) The supernatant of each condition was then collected to assess viral neutralization using the focus-forming assay titration protocol. Scale bars, 20 μm (A, B, D, and E).

The above date indicated that TNTs could mediate the infection of nonpermissive cells in coculture. We then asked whether the TNT-mediated route could also be used to enhance the spreading of the virus between permissive cells, in addition to the endocytic route. To this aim, we analyzed whether the virus was able to spread between permissive cells after blocking receptor-mediated entry (*23*). To impair binding to the ACE2 receptor, we used a neutralizing antibody that binds to the receptor-binding domain of the S protein [anti–SARS-CoV-2 human immunoglobulin G (IgG) C3 235]. Vero E6 cells infected with SARS-CoV-2 (MOI of 0.05) (donor cells) for 48 hours were incubated with anti–SARS-CoV-2 IgG C3 235 (10 μg/ml) at 37°C for 1 hour at 5% CO_2_, shown to neutralize the virus (fig. S8), before coculturing, in the presence of the antibody, with Vero E6 cells expressing mCherry (acceptor cells) to distinguish them from the infected donor population. After 24 hours of coculture in the presence of the anti-S neutralizing antibody, 42.9% of acceptor cells were positive for SARS-CoV-2 detected by anti-N immunostaining, and this percentage increased to 63.8% after 48 hours of coculture, compared to coculture control conditions (not incubated with the anti-S neutralizing antibody), where 95% at 24 hours and 96.8% at 48 hours of acceptor cells were positive for anti-N immunostaining, respectively (Fig. 4, A to C). As control, we challenged naïve Vero E6 cells with the supernatants of both the cocultures (incubated and not with the 235 antibody) (Fig. 4, D to F). While 100% of cells infected with the untreated supernatant were positive for SARS-CoV-2 (Fig. 4, D and F), no infection could be detected in the cells challenged with the treated supernatant (Fig. 4, E and F). Furthermore, the supernatants of each condition were collected to determine the virus concentration using the focus-forming assay (Fig. 4G).

**Fig. 5.**
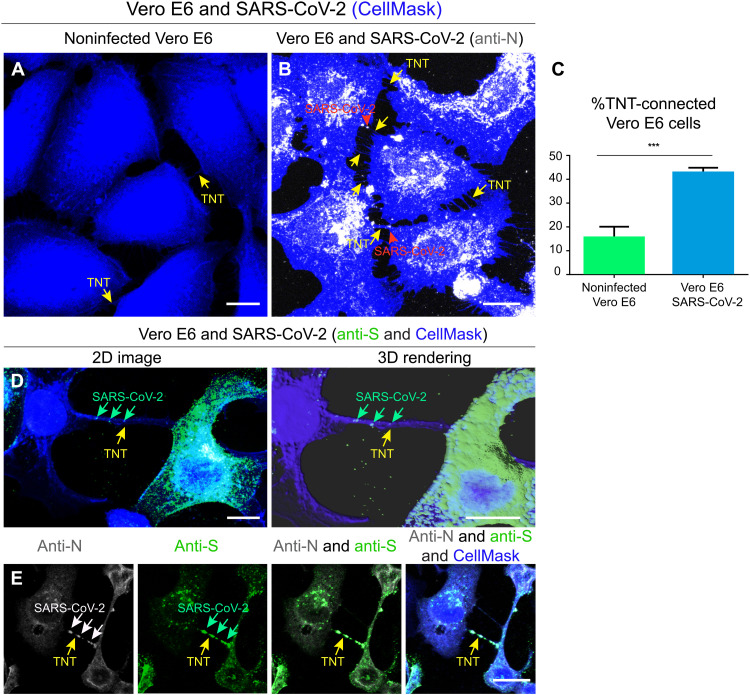
SARS-CoV-2 infection increases the number of TNTs between infected Vero E6 cells. (**A**) Confocal micrograph showing TNTs between noninfected Vero E6 cells. (**B**) Confocal micrograph showing TNTs between SARS-CoV-2–infected Vero E6 cells. Anti-N immunostaining is performed to detect SARS-CoV-2. Cellular cytoplasm and TNTs were labeled with CellMask Blue. The yellow arrows indicate TNTs between Vero E6 cells; the red arrowheads indicate the SARS-CoV-2 signal associated with TNTs. (**C**) Graph showing the percentage of TNT-connected cells between Vero E6 cells that are noninfected and SARS-CoV-2–infected. Mean percentage of TNT-connected Noninfected Vero E6 cells: 13.95% ± 2.46. Mean percentage of TNT-connected SARS-CoV-2–infected Vero E6 cells: 44.69% ± 1.96 (***P = 0.0006 for SARS-CoV-2–infected Vero E6 cells versus noninfected Vero E6 cells; *n* = 3). (**D**) Confocal micrograph and 3D rendering showing TNTs between SARS-CoV-2–infected Vero E6 cells; an anti-S immunostaining was performed to detect SARS-CoV-2. Cellular cytoplasm and TNTs were labeled with CellMask Blue. The yellow arrows indicate a TNT between infected Vero E6 cells; the green arrows indicate SARS-CoV-2 associated with a TNT. (**E**) Confocal micrograph showing TNTs between SARS-CoV-2–infected Vero E6 cells labeled with CellMask Blue. Anti-N (633) and anti-S (488) immunostaining was performed to detect SARS-CoV-2. The yellow arrows indicate a TNT between infected Vero E6 cells; the white and the green arrows indicate SARS-CoV-2 particles inside TNTs. Scale bars, 15 μm (A, B, and E) and 10 μm (C).

These data indicate that SARS-CoV-2 can also spread between permissive cells through a secretion-independent pathway. Consistent with this, the percentage of Vero E6 cells connected by TNTs substantially increased after 24 hours of SARS-CoV-2 infection (Fig. 5, A to C), and we could observe TNTs decorated with puncta positive for the N, S, or both antibodies (Fig. 5, B to E).

Last, consistent with the fact that the virus cannot enter neuronal cells via the classic endocytic pathway, the blocking antibody had no effect on neuronal cell infection in coculture (fig. S9, A to C). After 48 hours of coculture, we found no difference in the percentage of cells positive for SARS-CoV-2 detected by anti-N immunostaining, 57 and 51% of acceptor cells positive under the control condition or in the presence of neutralizing antibody, respectively (fig. S9, A and C). As control, we challenged naïve SH-SY5Y mCherry cells with the supernatants of both the cocultures (incubated or not with the blocking antibody). No infection was detected under either condition (fig. S9B). Overall, these data confirm that SARS-CoV-2 can spread from permissive to nonpermissive cells through a direct cell-to-cell contact, secretion-independent pathway.

### Cryo-EM reveals SARS-CoV-2 associated with TNTs

Although suggestive, the limited resolution of fluorescence microscopy cannot provide definitive information about the nature and structure of the viral particles being transmitted by TNTs. Neither can they discriminate whether the infectious particles were inside the lumen of the TNTs or on top of the TNT membrane. To answer these fundamental questions and overcome these limitations, we established CLEM, cryo-EM, and cryo-ET. These techniques allowed us to assess (in correlative mode) both SARS-CoV-2 and TNT architecture under the closest to native conditions.

**Fig. 6.**
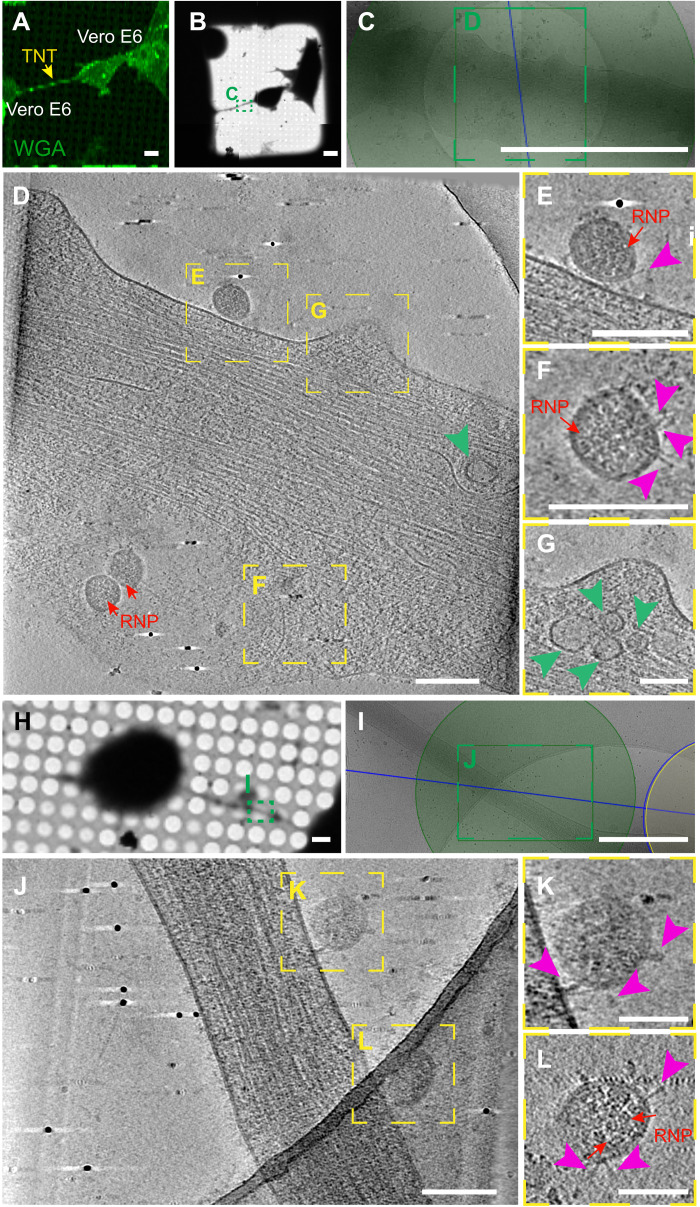
Cryo-CLEM reveal SARS-CoV-2 on TNTs between infected Vero E6 cells. (**A**) TNT-connected SARS-CoV-2–infected Vero E6 cells stained with wheat germ agglutinin (WGA) (green) and acquired by confocal microscopy (A), with low (**B**) and intermediate (**C**) magnification TEM. (**D**) Slices of tomograms of TNT in green dashed square in (C) showing the extracellular SARS-CoV-2 on top of the TNT connecting Vero E6 cells. (**E** to **G**) High-magnification cryo-ET slices corresponding to the yellow dashed squares in (D) showing SARS-CoV-2; RNPs and S proteins are observed. Pink arrowheads indicate the spike; red arrows point the RNPs. (E and F) High-magnification cryo-ET slices showing the extracellular virions on TNT. (G) High-magnification cryo-ET slices corresponding to the yellow dashed squares in (D). (G) The slices tomograms showed small vesicle compartments with a diameter of around 50 to 100 nm inside TNT. Green arrowheads point the vesicles. (**H** and **I**) Low (H) and intermediate (I) magnification of an electron micrograph of TNT-connected SARS-CoV-2–infected Vero E6 cells. (**J**) High-magnification cryo-ET slice corresponding to the green dashed rectangle in (I). (**K** and **L**) High-magnification cryo-ET slices showing the extracellular virions on TNT. Scale bars, 10 μm (A, B, and H), 2 μm (C), 150 nm (D to F and J), 100 nm (G, K, and L), and 1 μm (I).

Vero E6 cells were infected with SARS-CoV-2 (MOI of 0.05) and, 48 hours after infection, were seeded on cryo-EM grids. After having identified by fluorescence microscopy the exact location of TNTs connecting infected Vero E6 cells (Fig. 6A), the EM grids were cryo-fixed and analyzed by cryo-EM (Fig. 6). High-quality three-dimensional (3D) images using a 300-kV Titan Krios cryo–transmission electron microscope (TEM) revealed SARS-CoV-2 viral particles located on the surface of TNTs connecting two Vero E6 cells (Fig. 6, D to F and J to L, and movies S1 and S2). SARS-CoV-2 particles that decorated TNTs displayed a spherical enveloped morphology with an average diameter ranging from 50 to 100 nm, typical of a CoV (Fig. 6 and movies S1 and S2). In our tomograms, we could discern the S proteins that decorate the surface of the viral particles and the ribonucleoprotein (RNP) complexes organized inside the virus (Fig. 6, D to F and J to L, and movies S1 and S2) in accordance with recent cryo-EM data for the virus isolated from infected cells (*55*–*57*) and in situ cryo-ET of cryo-focused ion beam (cryo-FIB) in milled infected cells (*45*).

**Fig. 7.**
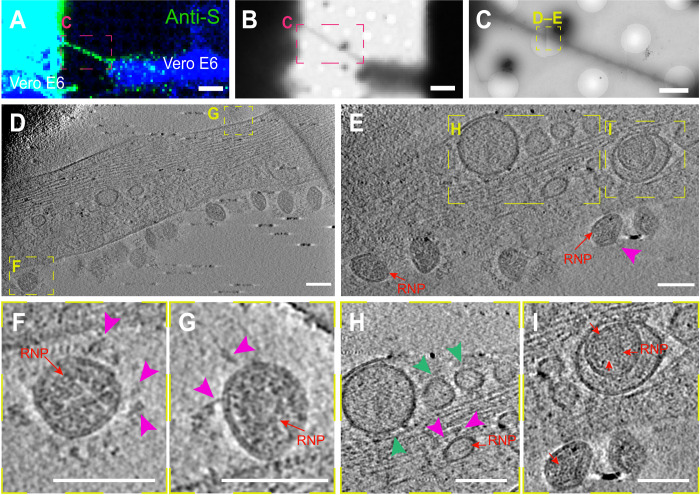
Correlative IF cryo-EM reveals SARS-CoV-2 localization in TNTs. (**A** to **H**) Cryo-EM grids were prepared using infected Vero E6 cells stained with anti-S antibody. (A) Confocal micrograph showing TNT connecting infected Vero E6 cells stained with anti-S antibody (green) and CellMask Blue (in pink dashed square). Low (B) and intermediate (C) magnification TEM of (A). (D and E) High-magnification cryo-ET slices of the yellow dashed square in (C) showing vesicular compartments in correspondence of anti-S signal and SARS-CoV-2 virions inside and on the TNT surface. (F and G) Enlargement of the high-magnification cryo-ET slices (D and E) and movie S6 showing SARS-CoV-2 virions on the TNT surface. (H) Enlargement of the high-magnification cryo-ET slices (D1) showing TNTs containing small vesicular structures (diameter of 50 to 100 nm) and a virus-like structure in which RNPs and S-like structures are observed. (**I**) Enlargement of the high-magnification cryo-ET slices (D1) showing virus-like structures inside a larger vesicle where we could discern the RNP. Pink arrowheads indicate S-like structures, red arrows point the RNPs, and green arrowheads indicate small vesicles. Scale bars, 15 μm (A and B), 2 μm (C), and 100 nm (D to H).

We also observed vesicular structures (average diameter of 50 to 100 nm) inside TNTs connecting infected Vero E6 cells (Fig. 6, D and G, green arrowhead; and movie S1). As the identification of structures inside TNTs is more challenging compared to their analysis at the TNT surface, to unequivocally demonstrate that these vesicular structures correspond to the virus and/or viral compartments, we set up a challenging correlative immunofluorescence (IF) cryo-EM protocol, making use of the anti-S antibody (see Methods) (Fig. 7, fig. S10, and movies S3 and S4). In correspondence with the fluorescent anti-S antibody signal in TNTs emerging from Vero E6 cells (Fig. 7A and fig. S10A), we could observe several mature virions decorating TNT surfaces with both spherical and ellipsoidal morphologies, as well as distinguish both RNPs and spikes (Fig. 7, D to G; fig. S10, D and G; and movies S3 and S4). We also observed multiple vesicular structures with a diameter of about 50 to 100 nm inside the TNT lumen (Fig. 7, E and H, and movie S3). In some instances, both RNP and/or S structures were recognizable (Fig. 7H; fig. S10, F and G; and movies S3 and S4). In addition, virus-like structures, where we could discern the RNP, and/or S-like structures were found inside larger vesicle in TNTs (Fig. 7, E and I; fig. S10, D and E; and movie S3). These diverse vesicular structures inside TNTs might include replicative complexes and mature virus, as both forms are present inside vesicles and can be transferred via TNTs. However, because the TNTs described here have an average diameter of more than 500 nm, we are at the resolution limits of the microscope (*58*, *59*), so we were not able to discriminate the precise structures of all the vesicular compartments found inside the nanotubes, compared with the clearer picture of mature virions observed outside the TNTs.

### Cryo-EM reveals viral compartments in TNTs between permissive and nonpermissive cells

**Fig. 8.**
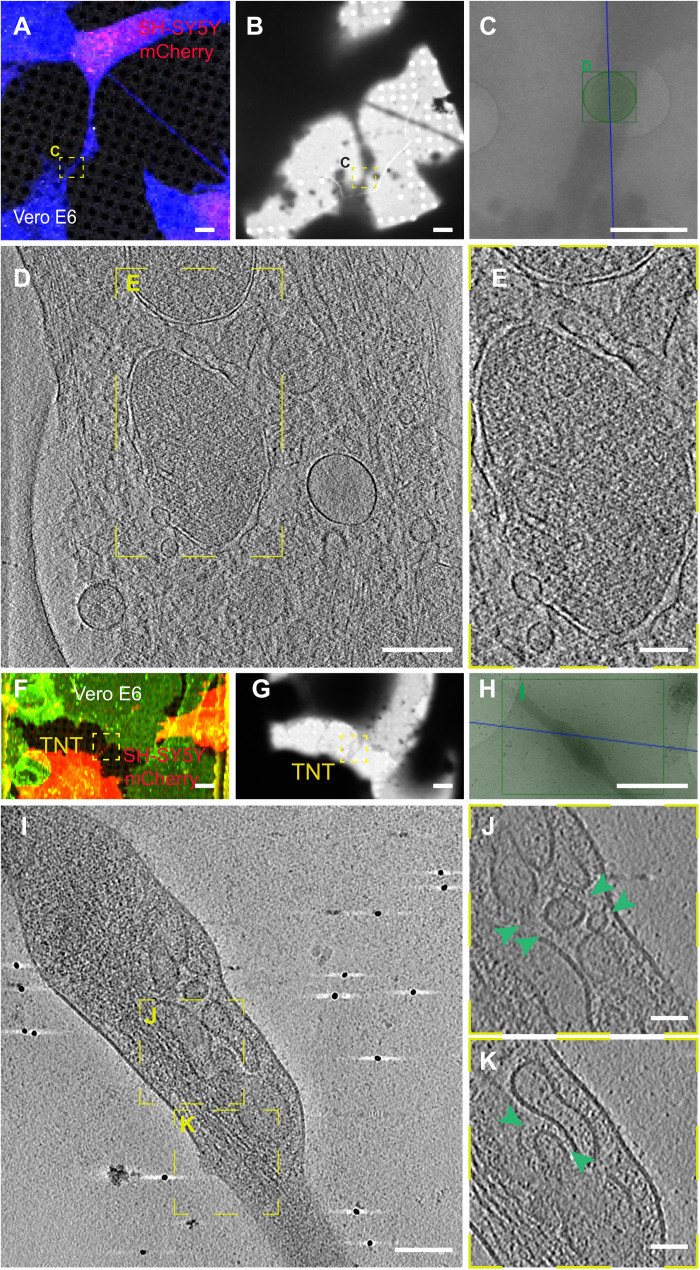
Ultrastructural analysis reveals SARS-CoV-2 compartments inside TNTs between permissive Vero E6 cells and nonpermissive SH-SY5Y neuronal cells. (**A**) Confocal micrographs showing a TNT connecting SARS-CoV-2–infected Vero E6 cells and SH-SY5Y mCherry cells stained with CellMask Blue. (**B**) Low and (**C**) intermediate magnification of an electron micrograph displaying TNT in (A). Green square in (C) corresponds to the high-magnification of cryo-ET slices in (**D**) showing a TNT containing vesicular compartments and DMVs. (**E**) Enlargement of cryo-ET slices in (D). (**F** to **K**) Cryo-EM grids were prepared using infected Vero E6 cells cocultured with SH-SY5Y mCherry cells and stained with WGA-488. (F) TNT between SARS-CoV-2–infected Vero E6 cells and SH-SY5Y mCherry cells acquired by confocal microscopy (F), with low (G) and intermediate (H) magnification TEM. (I) Slices of tomograms of TNT in the green rectangle in (H) showing vesicular compartments inside TNT. (J and K) High-magnification cryo-ET slices corresponding to the yellow dashed squares showing vesicular compartments inside TNT. The green arrowheads indicate the vesicles inside TNT. Scale bars, 2 μm (A to C and F to H), 200 nm (D), 300 nm (E), 100 nm (I), and 50 nm (J and K).

Next, to reveal the structure of the viral particles corresponding to the fluorescent labeling found in TNTs between permissive and nonpermissive cells, we applied the same cryo-CLEM techniques to the cocultures between Vero E6 and SH-SY5Y mCherry cells. Forty-eight hours after infection with SARS-CoV-2, Vero E6 cells were seeded on cryo-EM grids in coculture with SH-SY5Y mCherry cells (Fig. 8, A and F). Consistent with our previous data, we could detect TNTs between Vero E6 and SH-SY5Y mCherry (Fig. 8, A and F), and using the grid finders after vitrification, we could precisely identify the TNT positions and image them at the ultrastructural level using both 200-kV cryo-TEM equipped with Falcon 3 direct electron detectors (Thermo Fisher Scientific Glacios) (Fig. 8, A to E) and 300-kV Titan Krios cryo-TEM (Fig. 8, F to K). Notably, TNTs connecting infected Vero E6 cells and SH-SY5Y mCherry cells revealed the presence, inside the nanotubes, of membranous structures of various sizes resembling DMVs (Fig. 8, D and E, and movie S5), previously identified as the central hub for SARS-CoV-2 RNA synthesis by Klein and collaborators (*45*). Furthermore, as shown in the tomogram in Fig. 8I and in movie S6, the TNTs also contained many vesicular structures (Fig. 8, I to K, green arrow, and movie S6) similar to those observed inside TNTs between permissive cells (Figs. 6, D and G, and 7, D and H). Notably, we never observed DMVs and this crowding of vesicular structures inside TNTs between Vero E6 and SH-SY5Y mCherry cells in the absence of SARS-CoV-2 infection (fig. S11, A to D, and movie S7), where we could rather see isolated vesicles or organelles, as in the case of the mitochondrion shown in fig. S11D and movie S7. As SARS-CoV-2 replication is associated with proliferation of membranes and the presence of DMVs where viral replication takes place (*45*), consistent with our IF data showing the colocalization between N and nsp3 (Fig. 3B), it is likely that these structures represent viral replicative complexes being transferred to acceptor cells. Furthermore, by confocal microscopy, we observed TNTs between infected Vero E6 and SH-SY5Y mCherry cells and TNTs between infected Vero E6 containing particles stained with J2 antibody against dsRNA (fig. S12, A and B). In these cocultures, we also found TNTs between SH-SY5Y cells, which contained particles labeled with J2 antibody (fig. S12B). Considering 100 TNTs between infected Vero E6 and SH-SY5Y mCherry cells, we found that the 27% of them are positive for nsp3. These data indicate that some of the vesicles that we observed in TNTs might carry viral RNA and replicative vesicles.

In contrast with permissive cells, in neuronal cells, we did not observe virus on top of TNTs (Fig. 8, D and I). This difference could be explained by the presence of the ACE2 receptor, which is only expressed on the cell surface and TNT membranes of Vero E6 cells and not on SH-SY5Y cells (fig. S2). Several previous reports have described viruses of different families (*60*, *61*) on top of filopodia and/or cellular extensions, including SARS-CoV-2 (*45*, *62*, *63*). In the specific case of SARS-CoV-2, the nature of the membrane protrusions and whether they correspond to sites for viral budding and whether they allowed viral transfer were not addressed. Our report now presents evidence that TNTs are a route for the spreading of SARS-CoV-2.

We have also characterized TNTs connecting naïve Vero E6 cells and found that, similar to SH-SY5Y cells, they are mostly actin positive (fig. S13A). However they are made of single tubes (fig. S13B), differently from those found in control SH-SY5Y cells where most consisted of multiple individual tunneling nanotubes (iTNTs) (*39*). On the other hand, TNTs containing viral materials are always made by single and actin-rich tubes, as shown in Figs. 6 (D and J), 7D, and 8I.

## DISCUSSION

Patients with COVID-19 exhibit a range of neurological symptoms, suggesting that SARS-CoV-2 can invade the CNS (*64*). After an autopsy of the brains of patients with COVID-19, CoV RNA has been detected (*65*), and the olfactory mucosa has been suggested as a route of viral entry (*19*). SARS-CoV-2 is known to infect human host cells by binding ACE2, of which the expression is low in neuronal cells (*27*). Nonetheless, several studies have reported the presence of SARS-CoV-2 in human pluripotent stem cell and primary neurons (*5*, *66*). Thus, how SARS-CoV-2 could enter neuronal cells is still an open question. Several viruses, such as the influenza virus, HIV, and herpes simplex virus (*31*), can use TNTs to transfer their genomes to naïve cells, a mechanism of direct cell-to-cell communication that allows evasion of host immunity and to avoid pharmaceutical targeting (*67*). Here, we demonstrate that human neuronal SH-SY5Y cells, nonpermissive to SARS-CoV-2 through an exocytosis/endocytosis-dependent pathway (fig. S1), can be infected after being cocultured with permissive Vero E6 epithelial cells previously infected with SARS-CoV-2 (Fig. 1). Furthermore, our data using remdesivir and immunostaining for the viral replicative markers J2 and nsp3 support that SARS-CoV-2 is able to replicate once inside neuronal cells (Fig. 2 and fig. S5). By blocking the ACE2-mediated entry of the virus with a neutralizing antibody, we also demonstrate that SARS-CoV-2 can spread between permissive cells through a secretion-independent pathway. We speculate that TNTs accelerate the propagation of the infection, even between permissive cells. Because TNTs are dynamic transient structures (*28*) where actin is able to polymerize and depolymerize rapidly (i.e., 30 to 60 s) (*68*, *69*), the virus could potentially spread faster through TNTs than through other routes (*67*). Previous evidence indicated that upon interaction with cellular protrusions, viruses undergo rapid actin- and myosin II–mediated transport by “surfing” on the cell surface before reaching entry sites closer to the cell body (*60*). Moreover, TNTs may contain unconventional actin-based myosin motor proteins such as myosin Va (MyoVa) and MyoX (*28*, *70*). MyoVa has been suggested to mediate an actomyosin-dependent transport of endocytic vesicles in TNTs (*28*), while MyoX has been proposed as a major player in TNT formation (*71*).

Notably, SARS-CoV-2 infection resulted in an increase in the percentage of TNT-connected cells both between Vero E6 cells and between Vero E6 and SH-SY5Y cells (Fig. 5 and fig. S7). This evidence supports our hypothesis that SARS-CoV-2, similar to other viruses such as HIV (*30*, *72*), is an inducer of TNT formation, to facilitate its spreading between TNT-connected cells. SARS-CoV-2 might be able to induce TNT formation via several mechanisms. A recent publication has shown that SARS-CoV-2 infection induces a marked increase in filopodial protrusions, a process in which casein kinase II (CK2) plays a role (*63*). CK2 activity was significantly up-regulated in SARS-CoV-2–infected Vero E6 cells (*63*). CK2 might also be involved in the increase in TNTs, as it promotes actin polymerization and regulates the organization of the cytoskeleton (*73*). CK2 is known to phosphorylate myosin proteins at endocytic sites to drive actin polymerization (*74*). For example, Marburg virus hijacks the unconventional motor protein MyoX, which promotes filopodia formation and the traffic of the virus along them (*31*). We have previously shown that MyoX is a positive regulator of TNT formation in neuronal cells (*71*). It would be interesting to investigate whether MyoX is also involved in the TNT formation induced by SARS-CoV-2 and its movement along TNTs. In addition, Bouhaddou *et al.* (*63*) showed the activation of the p38 mitogen-activated protein kinase (MAPK) signaling pathway after SARS-CoV-2 cell infection. Activation of the p38 MAPK could also increase TNT formation (*75*). By confocal microscopy, we detected the viral proteins (S and N) and the replicative marker (J2 and nsp3) within TNTs, suggesting that TNTs could transfer viral materials. To better investigate how SARS-CoV-2 transfer through TNTs, we set up a challenging approach called CLEM, cryo-EM, and cryo-ET (*39*). These techniques allowed us to assess (in correlative mode) both SARS-CoV-2 and TNT architecture under the closest to native conditions. We found multiple SARS-CoV-2 virions (detected using an anti-S antibody) associated with the PM of TNTs formed between permissive cells (Figs. 6 and 7). We also observed the vesicular structures of different sizes in correspondence with the fluorescent signal of the virions inside TNTs. Similar viral vesicular structures and DMVs were present inside TNTs between permissive and nonpermissive cells (Fig. 8). Notably, we observed the virus on top of the TNTs formed between permissive cells and not in heterotypic coculture with neuronal cells. This discrepancy could be explained by the presence of the ACE2 receptor only on the TNT membranes derived from Vero E6 cells and not from SH-SY5Y cells. However, the lipid composition of the TNT membranes might also vary between different cell types. Because we observed that SARS-CoV-2 particles adhere to the surface of cell protrusions (i.e., TNTs) connecting two permissive cells, we hypothesize that SARS-CoV-2 might “surf” on the cell membrane. On the basis of our observations, the transfer of SARS-CoV-2 occurs via TNTs through both extracellular adhesion (i.e., surfing) and intracellular transport in agreement with what has been already shown for HIV (*30*). However, we cannot exclude that other mechanisms of direct cell-to-cell contact might be involved in SARS-CoV-2 spreading to uninfected cells. For example, HIV is able to not only “hijack” TNTs but also gap junctional communication to spread toxic signals to uninfected astrocytes (*76*). Klein *et al.* (*45*) described that SARS-CoV-2 virions remain attached to the cell surface after exocytosis due to the interaction of S protein with ACE2, suggesting that ACE2 might control not only virion entry but also its release into surrounding environments. Therefore, the virions we observed by cryo-CLEM and cryo-ET on top of TNTs could also be egressed from the cell. It is unclear whether TNTs could participate in virus exit. Although we did not observe virus exiting from TNTs, our data do not discriminate whether the virions that adhere on TNTs might be egressing and/or derive from the cell medium. Nonetheless, as we have previously shown that TNTs are different protrusions from filopodia (*39*, *53*), our observation might be different from the results published by Mendonça *et al.* (*77*) that show virus particles exiting through an extended protrusion using cryo–FIB and cryo-ET (*77*).

In conclusion, here, we show that SARS-CoV-2 is able to hijack TNTs to spread between connected cells, indicating that this intercellular route could contribute to the pathogenesis of COVID-19 and the spreading of the virus to nonpermissive neuronal cells. Within the limitation of the cellular model used, our report provides unprecedented structural information of SARS-CoV-2 by cryo-CLEM and cryo-ET and how it might use TNTs for spreading between permissive and nonpermissive cells to increase both viral tropism and infection efficiency. These results also pave the way to further investigations of the role of cell-to-cell communication in SARS-CoV-2 spreading to the brain in more physiological contexts (e.g., the potential role of TNTs in the spreading of the virus from the olfactory epithelium of the nasal cavity to the olfactory sensory neurons in the CNS and in contributing to the occurrence of the long COVID syndrome) and on alternative therapeutic approaches to impairing viral diffusion in addition to current investigations mainly focused on blocking S-receptor interactions.

## METHODS

### Cell lines and viruses

African green monkey kidney Vero E6 cell and colorectal adenocarcinoma human epithelial (Caco-2) cells were maintained at 37°C at 5% CO_2_ in Dulbecco’s minimum essential medium (DMEM) (Sigma-Aldrich) supplemented with 10% fetal bovine serum (FBS) and 1% penicillin/streptomycin. Human neuroblastoma (SH-SY5Y) cells were cultured at 37°C at 5% CO_2_ in RPMI 1640 (Euroclone), as well as 10% FBS and 1% penicillin/streptomycin. Mouse catecholaminergic neuronal cell line, CAD, were given by H. Laude (Institut National de la Recherche Agronomique, Jouy-en-Josas, France) and cultured at 37°C at 5% CO_2_ in Gibco Opti-MEM (Invitrogen), as well as 10% FBS and 1% penicillin/streptomycin.

The strain BetaCoV/France/IDF0372/2020 was supplied by the National Reference Centre for Respiratory Viruses hosted by Institut Pasteur (Paris, France) and headed by S. van der Werf. The human sample from which strain BetaCoV/France/IDF0372/2020 was isolated has been provided by X. Lescure and Y. Yazdanpanah from the Bichat Hospital, Paris, France. Moreover, the strain BetaCoV/France/IDF0372/2020 was supplied through the European Virus Archive goes Global (Evag) platform, a project that has received funding from the European Union’s Horizon 2020 Research and Innovation Programme under grant agreement no. 653316.

### Viral infection to identify SARS-CoV-2–permissive cells

To assess which cell lines were permissive to SARS-CoV-2 infection, the different cells were plated on a 96-multiwell plate and infected with an MOI from 10^−1^ to 10^−5^ in DMEM with 2% FBS. The cell lines used in this assay included Caco-2, CAD, SH-SY5Y, and Vero E6. All the cells were plated at a 60% confluence. The cells were incubated in infection medium for 3 days. At days 2 and 3 after infection, an aliquot of the supernatant from the higher MOI was collected for titration. At day 3 after infection, the monolayers were then fixed with 4% paraformaldehyde (PFA) for 45 min, and viral infection was visualized using an anti-N antibody.

### IF protocol for ImmunoSpot

After 45 min of incubation with 4% PFA, the monolayers were washed with phosphate-buffered saline (PBS) and incubated 5 min with 1× PBS–0.5% Triton X-100 at R.T. (room temperature); the cells were then washed and incubated for 10 min with 1× PBS–50 mM NH_4_Cl. After washing, 30 min of blocking was performed using 1× PBS–2% bovine serum albumin (BSA); the monolayers were incubated with the primary antibody, a polyclonal SARS-CoV anti-N IgG, provided by N. Escriou (Institut Pasteur, Paris) overnight at 4°C. After washing, the cells were then incubated with a goat anti-rabbit Alexa Fluor 488–conjugated antibody for 1 hour. After washing with 1× PBS to remove the unbound antibody, the IF was visualized using the Fluoro-X suite of a C.T.L. ImmunoSpot S6 image analyzer.

### Semisolid plaque assay

The aliquots of supernatant collected at day 2 and day 3 were used to assess viral production through a semisolid plaque assay. Each sample underwent 1:10 serial dilutions. A total of 250 μl of each dilution was used to infect a confluent monolayer of Vero E6 cells, in a 24-well multiwell plate, with a total of six wells per sample.

Viral absorption was allowed for 1 hour at 37°C, and a semisolid overlay, composed of 1× MEM, 10% FBS, and 0.8% agarose, was then added to the infection (250 μl per well). The cells were incubated at 37°C for 72 hours at 5% CO_2_. Last, the infected monolayers were fixed with 500 μl of 4% PFA for 30 min. Afterward, the PFA was removed, and the monolayers were then stained with crystal violet solution containing 2% PFA to evaluate the cytopathic effect. The reaction was stopped after 15 min, and residual crystal violet was removed through immersion in diluted bleach, followed by washing in water.

### Focus-forming assay

Vero E6 cells were plated in a 96-multiwell plate of 2 × 10^4^ cells per well. The monolayers were then infected with serial dilutions (1:10) of samples to be titrated. The infection was allowed at 37°C for 2 hours at 5% CO_2_. Afterward, the infection medium was removed, and a semisolid overlay composed of 1.5% carboxymethyl cellulose and 1× MEM was added to the monolayer. The cells were incubated at 37°C for 36 hours at 5% CO_2_ to allow foci formation. The monolayers were then fixed with 4% PFA; after 45 min, they were washed with PBS and incubated for 5 min with 1× PBS–0.5% Triton X-100 at R.T.; the cells were then washed again and incubated for 10 min with 1× PBS–50 mM NH_4_Cl. After washing, the cells were incubated 2 min in 0.05% PBS–Tween 20 and then incubated with the primary antibody, a polyclonal SARS-CoV anti-N IgG, provided by N. Escriou, Institut Pasteur, Paris (or alternatively with a human SARS-CoV-2 anti-S IgG provided by C. Planchais from the group of Hugo Mouquet Institut Pasteur, Paris), overnight at 4°C. After washing, the cells were then incubated with an anti-rabbit (or an anti-human) horseradish peroxidase–conjugated antibody for 1 hour. After washing with 1× PBS to remove the unbound antibody, the foci were visualized using a 3,3′-diaminobenzidine staining solution in PBS with 8% NiCl and washed three times with water to stop the reaction. The foci were then visualized and counted using the BioSpot suite of a C.T.L. ImmunoSpot S6 Image Analyzer.

### Lentiviral transduction

In transduction of SH-SY5Y and Vero E6 cells with a lentiviral vector expressing pCMV-mCherry, 600,000 SH-SY5Y cells and 400,000 Vero E6 cells were plated in 60-mm plates. After 24 hours, they were infected with 800 μl of LV-pCMV-mCherry. After 48 hours, cells expressing mCherry have been validated. In transduction of SH-SY5Y cells with a lentiviral vector expressing pCMV-H2B-GFP, 600,000 SH-SY5Y cells were plated in 60-mm plates. After 24 hours, they were infected with 800 μl of LV-pCMV-H2B-GFP. In transduction of SH-SY5Y cells with a lentiviral vector expressing pCMV-H2B-GFP, 600,000 SH-SY5Y cells were plated in 60-mm plates. After 24 hours, they were infected with 800 μl of LV-pCMV-H2B-GFP.

### SARS-CoV-2 infection of Vero E6 cells for coculture experiments and cryo-EM grids

A total of 1,000,0000 of donor Vero E6 cells were infected with an MOI of 0.05 in DMEM without FBS for 2 hours. Afterward, the infection medium was removed and substituted with fresh DMEM with 10% FBS. The cells were left in incubation at 37°C for 48 hours at 5% CO_2_. After that time, cells were trypsinized, centrifuged (1000 rpm for 10 min), counted, and seeded for the different experiments.

### Coculture preparation for SARS-CoV-2 transfer experiments and secretion test

A total of 1,000,0000 of donor Vero E6 cells were infected with an MOI of 0.05 in DMEM without FBS for 2 hours. Afterward, the infection medium was removed and substituted with fresh DMEM with 10% FBS. The cells were left in incubation at 37°C for 48 hours at 5% CO_2_. As acceptors were used, the nonpermissive SH-SY5Y cells and permissive Vero E6 cells stably transfected with a lentivirus expressed mCherry, according to the kind of experiment. The infected donors, as well as the acceptors cells, were trypsinized, centrifuged (1000 rpm for 10 min), counted, and cocultured on 24 glass coverslips at 37°C at 5% CO_2_ with a 1:1 ratio (50,000 donor–50,000 acceptor). After 24 and 48 hours, cocultures were washed with 0.01% trypsin to remove excess of virus on top of the cell membrane and fixed for 30 min with 4% PFA, and then we proceed processing the cocultures for immunostaining of anti-N and anti-S. After the immunostaining, cells were stained with the HCS CellMask Blue Stain (1:300; Invitrogen) in 1× PBS for 30 min and then mounted. Images were acquired on an LSM 700 confocal microscope (Zeiss) with a 40× objective.

After image acquisition, the number of acceptor cells, which had received SARS-CoV-2, identified by the anti-N and/or anti-S immunostaining was quantified. Briefly, after image acquisition, the number of acceptor cells, which had received SARS-CoV-2, identified by the anti-N and/or anti-S immunostaining was semiautomatedly quantified with the open-source software Icy (http://icy.bioimageanalysis.org/).

To evaluate the possibility of SARS-CoV-2 transfer from donor to acceptor cells mediated by secretion, the supernatants from SARS-CoV-2–infected Vero E6 cells were collected, centrifuged at 1000 rpm for 10 min to remove floating cells, and added on acceptor cells: SH-SY5Y mCherry. After 24 and 48 hours, acceptor cells were washed with 0.01% trypsin and fixed with 4% PFA at R.T. for 30 min. After image acquisition, acceptor cells were counted for the presence of SARS-CoV-2 signal. Secretion test was performed in parallel to all the coculture experiments performed in this study by following the same protocol. In addition, the supernatants from donor-infected cells were used to assess viral production by focus-forming assay titration protocol.

### IF labeling

Cells were fixed in 4% PFA for 30 min, quenched with 50 mM NH_4_Cl for 15 min, permeabilized with 0.5% Triton X-100 for 5 min in 1× PBS, and blocked with 1× PBS containing 2% BSA (w/v) for 1 hour. Cells were then incubated with primary antibody dissolved in 2% BSA in 1× PBS. The primary antibody used were the following: a rabbit anti-N (1:500; a gift from N. Escriou, Institut Pasteur, Paris) overnight, an anti-human spike (1:100; H2-162; produced by C. Planchais from the group of Hugo Mouquet Institut Pasteur, Paris) overnight, an anti-dsRNA monoclonal antibody J2 (1:50; RNT-SCI-10010200, Jena Bioscience) overnight, an anti-sheep nsp3 (1:200; MRC PPU Reagents) overnight, and anti-rabbit Giantin (1:500; BioLegend) overnight.

The day after, cells were thoroughly washed and incubated for 40 min with an anti-rabbit Alexa Fluor 633–conjugated secondary antibody (Invitrogen), an anti-human Alexa Fluor 488–conjugated secondary antibody (Invitrogen), goat anti-mouse Alexa Fluor 633–conjugated secondary antibody (Invitrogen), donkey anti-sheep IgG (H + L) Alexa Fluor 488–conjugated secondary antibody (Invitrogen), and anti-rabbit Alexa Fluor 488–conjugated secondary antibody (Invitrogen) at 1:500 in 2% BSA (w/v) in 1× PBS. Cells were then carefully washed in 1× PBS and labeled with the HCS CellMask Blue Stain (1:300; Invitrogen) in 1× PBS for 30 min and then mounted. For anti-ACE2 antibody (PA5-20046, Thermo Fisher Scientific) immunostaining, cells were fixed in 4% PFA for 10 min, quenched with 50 mM NH_4_Cl for 15 min, and blocked with 1× PBS containing 2% BSA (w/v) for 1 hour. Cells were then incubated with primary antibody overnight dissolved in 2% BSA in 1× PBS. The day after, cells were thoroughly washed and incubated for 40 min with an anti-rabbit Alexa Fluor 488–conjugated secondary antibody (Invitrogen) at 1:500 in 2% BSA (w/v) in 1× PBS. Cells were then carefully washed in 1× PBS and labeled with the HCS CellMask Blue Stain (1:300; Invitrogen) in 1× PBS for 30 min and then mounted.

For KDEL (SPA-827, Enzo Life Sciences) and nsp3 immunostaining, cells were fixed in 4% PFA for 30 min, quenched with 50 mM NH_4_Cl for 15 min, blocked, and permeabilized with 1× PBS containing 0.0075% saponin and 0.01% gelatin for 30 min. Cells were then incubated with primary antibody KDEL (1:100) and nsp3 (1:300) overnight and dissolved in 0.0075% saponin and 0.01% gelatin in 1× PBS. The day after, cells were thoroughly washed and incubated for 40 min with an anti-goat anti-mouse Alexa Fluor 633–conjugated secondary antibody (Invitrogen) for KDEL and donkey anti-Sheep IgG (H + L) Alexa Fluor 488–conjugated secondary antibody (Invitrogen) for nsp3. Cells were then carefully washed in 1× PBS and labeled with the HCS CellMask Blue Stain (1:300; Invitrogen) in 1× PBS for 30 min and then mounted.

For microtubule and actin staining, cells were prewarmed with PHEM buffer [60 mM Pipes (pH 6.9), 25 mM Hepes, 10 mM EGTA, and 2 mM MgCl_2_ in H_2_O] before fixing with 4% PFA and 0.05% glutaraldehyde (GA) in PHEM for 30 min at 37°C. Cells were then incubated in a 50 mM NH_4_Cl solution for 15 min at R.T. Cells were further permeabilized with 0.1% Triton X-100 in 1× PBS for 2 min. After three washes with 1× PBS, cells were blocked using 2% BSA in 1× PBS for 30 min. Cells were then incubated for 1 hour with mouse anti–α-tubulin antibody (T9026, Sigma-Aldrich) diluted 1:500 in 2% BSA in 1× PBS. Washed cells were then incubated with goat anti-mouse Alexa Fluor 488 nm (Invitrogen) diluted 1:500 in blocking solution for 40 min. For F-actin detection, cells were stained with 0.6 μM rhodamine-phalloidin in PBS for 20 min.

### Coculture preparation for SARS-CoV-2 transfer experiments in the presence of neutralizing antibody

The viral stock of 1 × 10^5^ to 5 × 10^5^ focus-forming units (FFU)/ml used to infect Vero E6 cells was incubated at 37°C for 1 hour at 5% CO_2_ with three different concentrations of IgG C3 235 (1, 10, and 100 μg/ml) to determinate the minimal concentration of antibody sufficient to achieve its neutralization. The neutralized viral stock was then used to infect monolayers of Vero E6 cells for 48 hours. Viral production was then assessed by titration of the supernatant by focus-forming assay. Both 100 and 10 μg/ml concentration of antibody were enough to elicit complete neutralization of the viral stock, resulting in no sign of viral production. Therefore, a concentration of 10 μg/ml was chosen as the minimal concentration to investigate direct cell-to-cell transfer of SARS-CoV-2 in Vero E6 cells. Vero E6 donor cells, infected as previously described, were put in coculture, in a 1:1 ratio, with Vero E6 mCherry acceptors and SH-SY5Y mCherry acceptor cells in the presence of a SARS-CoV-2–neutralizing antibody. Briefly, infected donors were trypsinized and counted. They were then diluted at a concentration of 5 × 10^5^ cells/ml in DMEM with 5% FBS, containing a concentration of anti–SARS-CoV-2 IgG C3 235 (10 μg/ml; produced by C. Planchais from the group of Hugo Mouquet Institut Pasteur, Paris), which has been proved to be sufficient to elicit complete neutralization for a viral concentration of 1 × 10^5^ to 5 × 10^5^ FFU/ml. Donor cells were incubated in the presence of the antibody at 37°C for 1 hour at 5% CO_2_. Afterward, donor cells were cocultured at a ratio of 1:1 with Vero E6 mCherry and SH-SY5Y mCherry acceptor cells in DMEM 5% with FBS, with the aforementioned neutralizing antibody (10 μg/ml). The cocultures were incubated at 37°C for 24 and 48 hours at 5% CO_2_. Then, cocultures were fixed in 4% PFA for 30 min and immunostained for the anti-N (protocol described above) and with the HCS CellMask Blue Stain (1:300; Invitrogen) for 30 min. Images were acquired on an LSM 700 confocal microscope (Zeiss) with a 40× objective. After image acquisition, the number of acceptor cells, which had received SARS-CoV-2, identified by the anti-N immunostaining was quantified by the Icy software as before. In parallel, the supernatant of each condition was then collected to assess viral neutralization using focus-forming assay titration protocol. For the secretion test, performed in parallel with the coculture, an aliquot of the supernatant from the donor was incubated with anti–SARS-CoV-2 IgG C3 235 (10 μg/ml) for 1 hour at 37°C, to neutralize the viral particles, present in the supernatant. In parallel, another aliquot was left untreated for comparison. The supernatants were then added on top of acceptors cells. Afterward, we proceed for the analysis as before mentioned.

### Coculture preparation for SARS-CoV-2 transfer experiments in the presence of remdesivir

To determine the minimal concentration of remdesivir (Interchim, B60DF0) sufficient to block SARS-CoV-2 replication, naïve Vero E6 mCherry cells were preincubated for 1 hour at 37°C, with three different concentrations of remdesivir (3, 30, and 40 μM). They were then maintained in the presence of an inhibitor and were infected with SARS-CoV-2 at an MOI of 0.05. After 48 hours, SARS-CoV-2–infected Vero E6 cells were stained using an anti-dsRNA J2 antibody and anti-S antibodies to detect SARS-CoV-2 particles. A total of 30 μM was chosen as the minimal concentration to investigate whether the viral signal observed in the neuronal acceptor cells corresponds to de novo replicated virus. Infected Vero E6 donor cells were placed in coculture with SH-SY5Y mCherry acceptors pretreated at 37°C for 1 hour at 5% CO_2_. The cocultures were maintained in the presence of the inhibitor. After 48 hours, cocultures were fixed and immunostained with anti-S and anti-dsRNA J2 antibodies to detect SARS-CoV-2 particles. For the secretion test, SH-SY5mCherry and Vero E6 mCherry cells were preincubated or not with the remdesivir for 1 hour and then were maintained in the presence of the inhibitor and challenged with the supernatant of infected cells for 48 hours. Images were acquired on an LSM 700 confocal microscope (Zeiss) with a 40× objective. After image acquisition, the number of acceptor cells, which had received SARS-CoV-2, identified by anti-S and J2 immunostaining, was quantified using Icy software as before.

### TNT counting

For quantification of TNT-connected cells, Vero E6 cells infected (as described before) and not infected were trypsinized and counted; 50,000 cells were plated on 24 glass coverslips. For quantification of TNT-connected cells between Vero E6 and SH-SY5Y mCherry cells infected or not that were trypsinized and counted, 50,000 cells of Vero E6 and 50,000 cells of SH-SY5Y mCherry cells were plated on 24 glass coverslips. After 24 hours, cells were fixed (15 min at 37°C at 2% PFA, 0.05% GA, and 0.2 M Hepes in 1× PBS and then additionally fixed for 15 min in 4% PFA and 0.2 M Hepes in 1× PBS). Cells were carefully washed in 1× PBS, labeled for 20 min at R.T. with a solution (3.3 μg/μl) of wheat germ agglutinin (WGA) Alexa Fluor 488-nm conjugate (Invitrogen) in 1× PBS, washed again, and mounted. The whole cellular volume was imaged by acquiring 0.45-μm *z* stacks with an inverted confocal microscope (Zeiss LSM 700) using ZEN software. TNT-connected cells, connected by straight WGA-labeled structures that do not touch the substrate, were manually counted by Icy software using the semiautomatized TNT counting tool as previously described (*53*, *78*). The 3D renderings of TNTs were performed using Imaris software.

### Cell preparation for cryo-EM

Carbon-coated gold TEM grids (NH2A R2/2, QUANTIFOIL) were glow-discharged at 2 mA and 1.5 × 10^−1^ to 1.8 × 10^−1^ mbar for 1 min in an ELMO (Cordouan) glow discharge system. Grids were sterilized under ultraviolet three times for 30 min at R.T. and then incubated at 37°C at complete culture medium for 2 hours. A total of 200,000 infected Vero E6 cells (48 hours after infection) were counted and seeded on cryo-EM grids positioned in 35-mm Ibidi μ-Dish (BioValley, France). For coculture, 100,000 infected Vero E6 cells (48 hours after infection) were cocultured with 100,000 SH-SY5Y mCherry on cryo-EM grids in 35-mm Ibidi μ-Dish (BioValley, France). After 24 hours, cells resulted in three to four cells per grid square. Before chemical and cryo-plunging freezing, cells were labeled with WGA–Alexa Fluor 488 (1:300 in PBS) for 5 min at 37°C. For cryo-CLEM, cells were chemically fixed in 2% PFA and 0.05% GA in 0.2 M Hepes for 15 min followed by fixation in 4% PFA in 0.2 M Hepes for 15 min and were kept hydrated in 1× PBS buffer before vitrification.

For cryo-CLEM using the anti-S primary antibody, cells were fixed with 4% PFA for 30 min at 37°C, quenched with 50 mM NH_4_Cl for 15 min, and blocked with 1× PBS containing 2% BSA (w/v) for overnight at 4°C. Cells were labeled with an anti-human Alexa Fluor 488–conjugated secondary antibody (Invitrogen) at 1:500 and labeled with the HCS CellMask Blue Stain (1:300; Invitrogen). For cell vitrification, cells were blotted from the back side of the grid for 10 s and rapidly frozen in liquid ethane using a Leica EM GP system as we performed before (*39*).

### Cryo-ET data acquisition and tomogram reconstruction

The cryo-EM data were collected from different grids at the Nanoimaging Core Facility of the Institut Pasteur using a Thermo Fisher Scientific 300-kV Titan Krios G3 cryo-TEMs equipped with a Gatan energy filter bioquantum/K3. Cryo-ET software from Thermo Fisher Scientific was used to acquire the data. Tomograms were acquired using a dose-symmetric tilt scheme (*79*); a ±60° tilt range with a tilt step 2 was used to acquire the tilt series. Tilt images were acquired in counting mode with a calibrated physical pixel size of 3.2 Å and total dose over the full tilt series of 3.295 *e*^−^/Å^2^ and dose rate of 39,739 *e*^−^ per pixel per second with an exposure time of 1 s. The defocus applied was in a range of −3- to –6-μm defocus.

The tomogram showed in Fig. 8D was performed on Thermo Fisher Scientific Glacios 200-kV cryo-TEM equipped with Falcon 3 direct electron detectors. Tilt series were recorded using Cryo-ET software (Thermo Fisher Scientific) in counting mode and an angular range of −60° to +60°, with a calibrated physical pixel size of 3.2 Å and a total dose over the full tilt series of 3.49 *e*^−^/Å^2^ and dose rate of 42.16 *e*^−^ per pixel per second and 3.49 *e*^−^/Å^2^ with 1-s exposure time and 70-μm objective apertures. The defocus applied was in a range of −3-μm defocus.

The tomograms were reconstructed using IMOD (eTomo). Final alignments were done using 10-nm fiducial gold particles coated with BSA (BSA Gold Tracer, Electron Microscopy Sciences). Gold beads were manually selected and automatically tracked. The fiducial model was corrected in all cases where the automatic tracking failed. Tomograms were binned two times corresponding to a pixel size of 0.676 nm for the Titan and 0.6368 nm for the Glacios, and SIRT (simultaneous iterative reconstruction technique)–like filter (*15*) option in eTomo was applied. For visualization purposes, the reconstructed volumes were processed by a Gaussian filter. The cryo-ET slice in fig. S11D is obtained by a collage of two different cryo-slices of the same tomogram.

### Optical resolution of the Titan Krios microscope

The optical resolution limit of an electron microscope of the Titan Krios class is 1.2 Å. This resolution has recently been achieved by two groups (*58*, *59*). Nevertheless, because of the nature of cryo-EM, where we have to use a limited electron dose in every image to prevent damage of the structure beyond the resolution we like to achieve, we have to record many copies of the same protein and apply extensive image processing procedures to classify and average different particle projections together to finally end up with a high-resolution structure. This method is called the single-particle analysis and has become a standard procedure in cryo-EM. In cryo-ET of cells, we are dealing with objects that are never alike: Each cell is different. We can therefore not apply any averaging image processing in this case. If one would be interested in resolving the viral spike of SARS-CoV-2 inside the native cell, then a method of subtomographic averaging can be used; however, our biological question in this paper does not allow such an approach. We are therefor left with the resolution of a single low-dose tomogram. Here, the thickness of the sample plays a decisive role. Thicker samples will require more dose to provide enough signal on the detector to still provide an image. While we are tilting to 60°, the sample will additionally get thicker, limiting the signal even more. Hence, for our experiments where the TNTs are of notable thickness, the signal and, thus, resolution in the final tomograms will be limited. Last, the nature of cryo-ET with discrete tilt steps in only one α-tilt direction causes the resolution in a single tomogram to be anisotropic in *X*, *Y*, and *Z*. For estimating the resolution, we can only guess on the basis of the structures we can recognize. A good estimate would be between 1- and 4-nm resolution.

### Statistical analysis

All column graphs and statistical analysis were performed using the GraphPad Prism version 7 software. Unpaired *t* test was applied for comparisons of two conditions presented inFigs. 1 and 5. For more than two groups, statistical significance was assessed by a one-way analysis of variance (ANOVA) with Tukey correction in Fig. 4C. Quantifications were done blind. Quantitative data were depicted as (±SEM) mean SD.

The graph in Fig. 1H shows the percentage of N transfer in coculture at 24 and 48 hours. The mean percentage of N transfer in coculture at 24 and 48 hours is 36.47% ± 3.96 and 62.56% ± 8.28, respectively (*P = 0.0468 for coculture at 48 hours versus coculture at 24 hours; *n* = 3). The graph in Fig. 1L shows the percentage of S transfer in coculture at 24 and 48 hours. The mean percentage of S transfer in coculture at 24 and 48 hours is 21.84% ± 5.09 and 42.44% ± 4.38, respectively (*P = 0.0374 for coculture at 48 hours versus coculture at 24 hours; *n* = 3). The graph in Fig. 4C shows the percentage of N transfer in coculture at 24 and 48 hours treated and not with the neutralizing antibody. The mean percentage of N transfer in coculture at 24-hour control is 95.45% ± 4.29, and that of N transfer in coculture at 24 hours plus neutralizing antibody is 42.91 ± 4.55; **P = 0.0018 for coculture at 24-hour control versus coculture at 24 hours plus neutralizing antibody. The mean percentage of N transfer in coculture at 48-hour control is 96.88% ± 3.12, and that of N transfer in coculture at 48 hours plus neutralizing antibody is 63.90 ± 1.99; *P = 0.0104 for coculture at 48-hour control versus coculture at 48 hours plus neutralizing antibody. *P* = 0.9914 [not significant (ns)] for coculture at 24-hour control versus coculture at 48-hour control. *P = 0.0122 for coculture at 24-hour control versus coculture at 48 hours plus neutralizing antibody. **P = 0.0016 for coculture at 24-hour control antibody versus coculture at 48 hours plus neutralizing antibody. *P = 0.0496 for coculture at 24 hours plus neutralizing antibody versus coculture at 48 hours plus neutralizing antibody. The mean percentage of N transfer in secretion at 24-hour control is 100% ± 0, and that of N transfer in coculture at 24 hours plus neutralizing antibody is 0 ± 0; ***P = 0.0005 for coculture at 24-hour control versus coculture at 24 hours plus neutralizing antibody. The mean percentage of N transfer in secretion at 48-hour control is 80% ± 10, and that of N transfer in coculture at 48 hours plus neutralizing antibody is 0 ± 0; ***P = 0.0008 for coculture at 48-hour control versus coculture at 48 hours plus neutralizing antibody. The graph in Fig. 5C shows the percentage of TNT-connected cells between noninfected Vero E6 and SARS-CoV-2–infected cells. The mean percentage of TNT-connected noninfected Vero E6 is 13.95% ± 2.46. The mean percentage of TNT-connected SARS-CoV-2–infected Vero E6 cells is 44.69% ± 1.96 (***P = 0.0006 for Vero E6 SARS-CoV-2 versus noninfected Vero E6; *n* = 3). PCC was used to quantify colocalization between anti-S and anti-N. Twenty cells were considered. PCC was calculated using JACoP plugins in Fiji. The length of the TNTs was measured using Fiji software. The TNTs positive for anti-nsp3 puncta have been counted manually using Fiji software. The graph in fig. S5C shows the percentage of S transfer in 48-hour coculture (control and treated with the inhibitor). The mean percentage of S transfer in coculture control is 46.2% ± 1.7, and that of S transfer in coculture plus inhibitor is 15.3% ± 2.1 (**P = 0.0075 for coculture plus inhibitor versus coculture control). The mean percentage of J2 transfer in coculture control is 43.2 ± 2.6, and that of J2 transfer in coculture plus inhibitor is 17.2% ± 0.2 (*P = 0.0101 for coculture plus inhibitor versus coculture control). The graph in fig. S7C shows the percentage of TNT-connected cells between noninfected Vero E6 and SH-SY5Y cells and TNT-connected cells between noninfected Vero E6 and SH-SY5Y cells. The mean percentage of TNT-connected Vero E6 and noninfected SH-SY5Y cells is 35% ± 2.02. The mean percentage of TNT-connected infected Vero E6 and SH-SY5Y cells is 61.75% ± 6.27 (*P = 0.0154 for coculture SARS-CoV-2–infected versus coculture noninfected; *n* = 3). The graph in fig. S9 shows the percentage of N transfer in coculture at 48 hours of treated or not with neutralizing antibody. The mean percentage of N transfer in coculture control is 57.01% ± 3.95, and that of N transfer in coculture plus neutralizing antibody is 50.92 ± 3.55 [*P* = 0.3154 (ns) for coculture control versus coculture plus neutralizing antibody; *n* = 3].

## Notes

View/request a protocol for this paper from *Bio-protocol*.
