# Supplementary material for: Tunneling nanotubes provide a route for SARS-CoV-2 spreading
Source: Sci Adv. 2022 Jul 20;8(29):eabo0171. doi: 10.1126/sciadv.abo0171 (PMC9299553; doi:10.1126/sciadv.abo0171)
Supplement: Supplementary file 1 — Figs. S1 to S13 [file sciadv.abo0171_sm.pdf]

Science Advances
AAAS

Supplementary Materials for

Tunneling nanotubes provide a route for SARS-CoV-2 spreading

Anna Pepe *et al.*

Corresponding author: Chiara Zurzolo, chiara.zurzolo@pasteur.fr

Sci. Adv. **8**, eabo0171 (2022)
DOI: 10.1126/sciadv.abo0171

The PDF file includes:

Figs. S1 to S13
Legends for movies S1 to S7

Other Supplementary Material for this manuscript includes the following:

Movies S1 to S7

Fig. S1

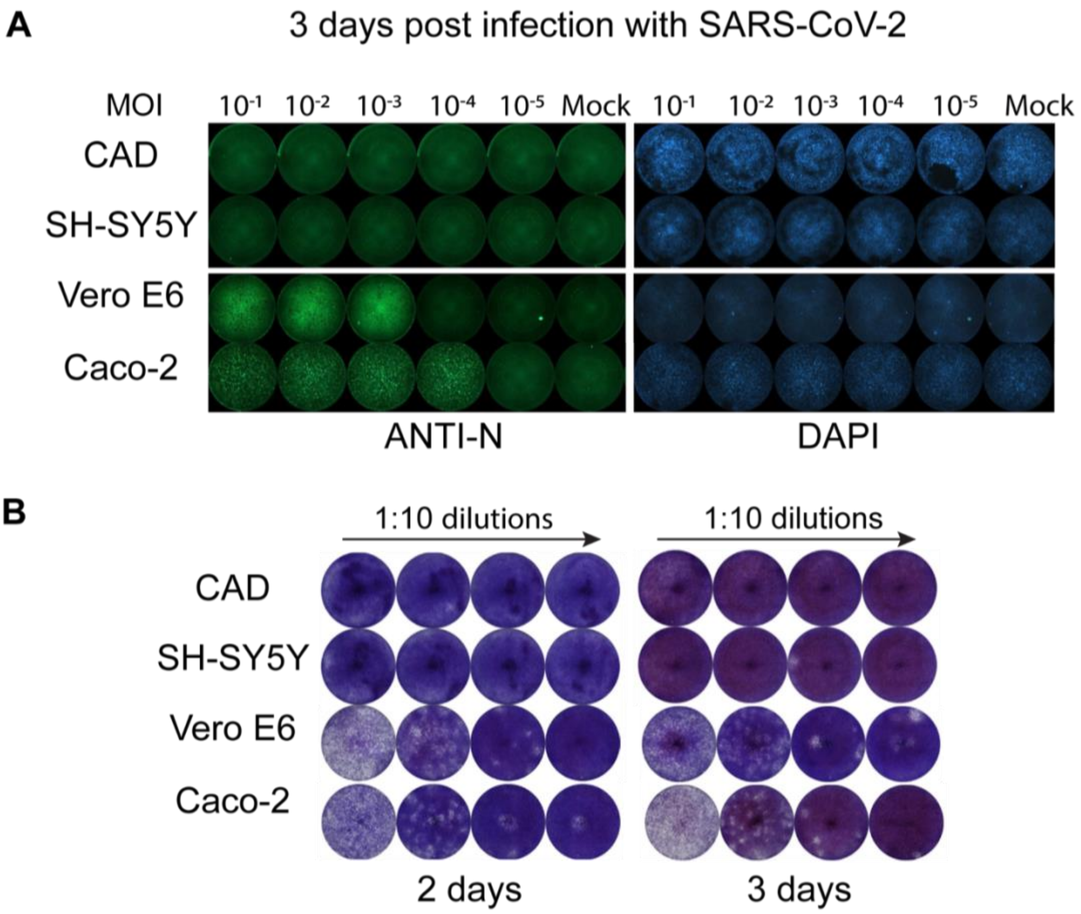

Fig. S1. Screening of cell lines for susceptibility to SARS-CoV-2. (A) CAD, SH-SY5Y, Vero E6, Caco-2 cells were plated on a 96 multi-well plate and infected with SARS-CoV-2 MOI from 10^{-1} to 10^{-5} in DMEM 2% FBS. At 3 days post infection the monolayers were fixed with 4% PFA and viral infection was visualized using an anti-N immunostaining and DAPI to stain the nuclei and then visualized using the Fluoro-X suite of a C.T.L. Immunospot® S6 Image Analyzer. (**B)** At day 2 and 3 post infection of the cells (CAD, SH-SY5Y, Vero E6, Caco-2), an aliquot of the supernatant from the higher MOI was collected for titration.

Fig. S2

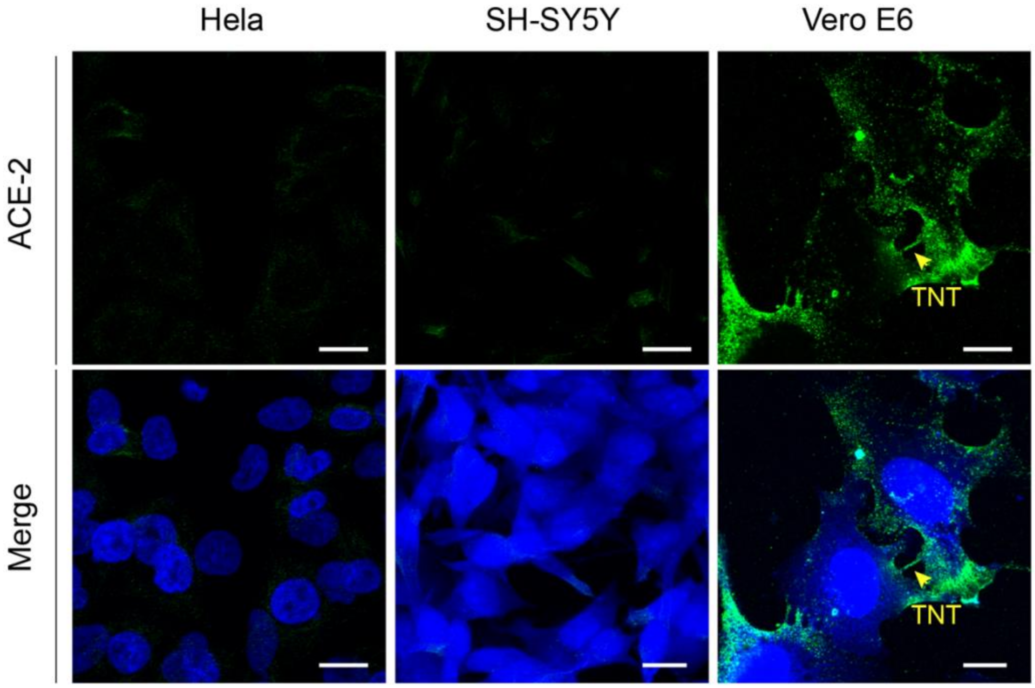

Fig. S2. ACE2 receptor expression. Confocal micrograph displaying Hela, SH-SY5Y and Vero E6 cells labeled with an anti-ACE2 antibody (green) and cell mask blue. Scale bars: 15µm.

Fig. S3

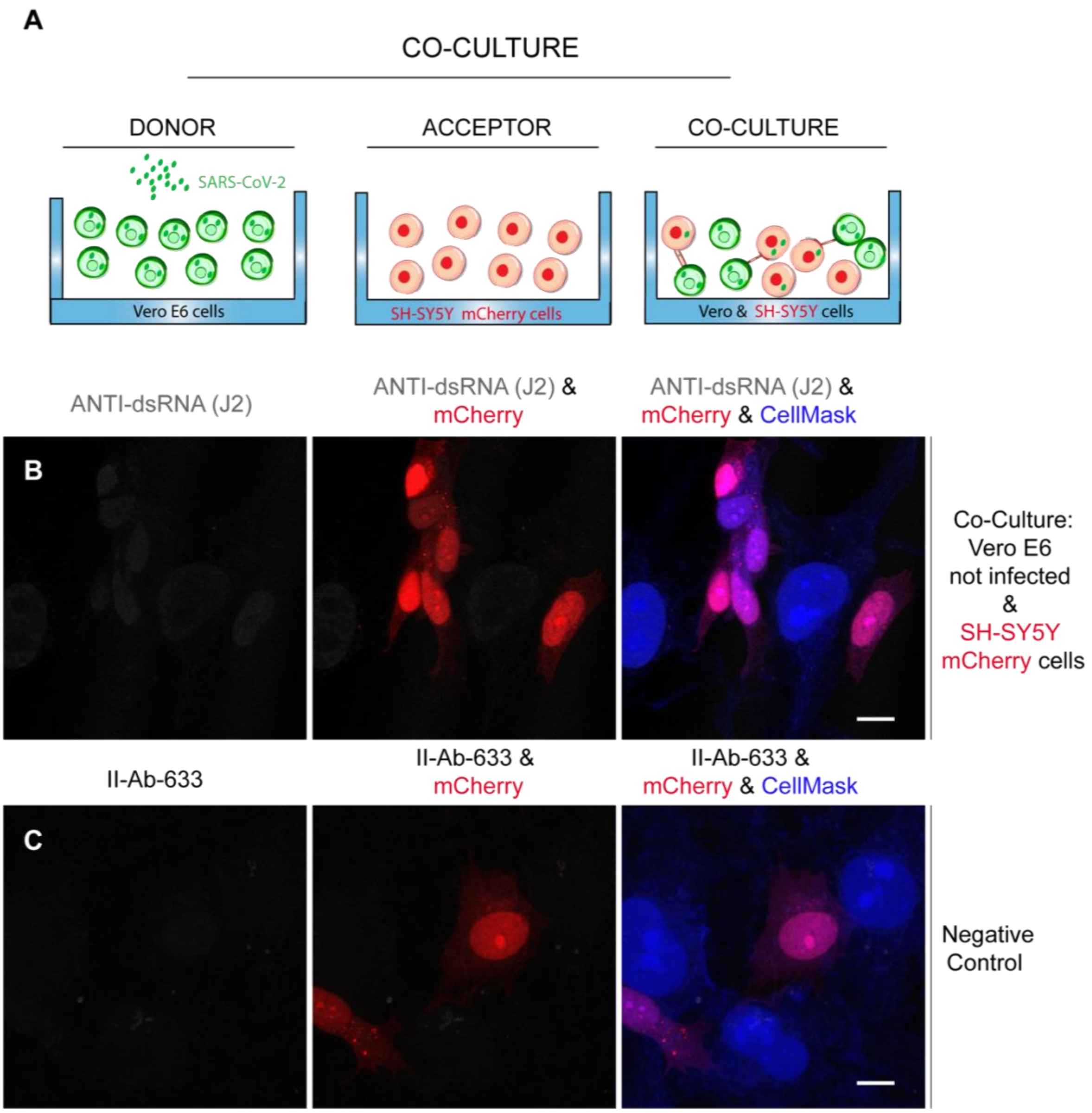

Fig. S3. Co-culture pipeline between infected Vero E6 cells and SH-SY5YmCherry cells and co-culture between not-infected Vero E6 cells and SH-SY5YmCherry cells. (A) Description of co-culture experiments: Donor Vero E6 cells were infected with SARS-CoV-2 MOI of 0.05 for 48h. After 48h, donor cells were co-cultured with the acceptor SH-SY5YmCherry cells and incubate for additional 24h and 48h before to be fixed. (**B**) Confocal micrographs showing not infected Vero E6 cells co-cultured with SH-SY5YmCherry cells. The co-culture was immunostained with J2 antibody and cell mask blue. (**C**) Negative control. The co-culture was immunostained with the secondary antibody conjugated with 633 fluorochrome and cell mask blue. Scale bars: 10µm.

Fig. S4

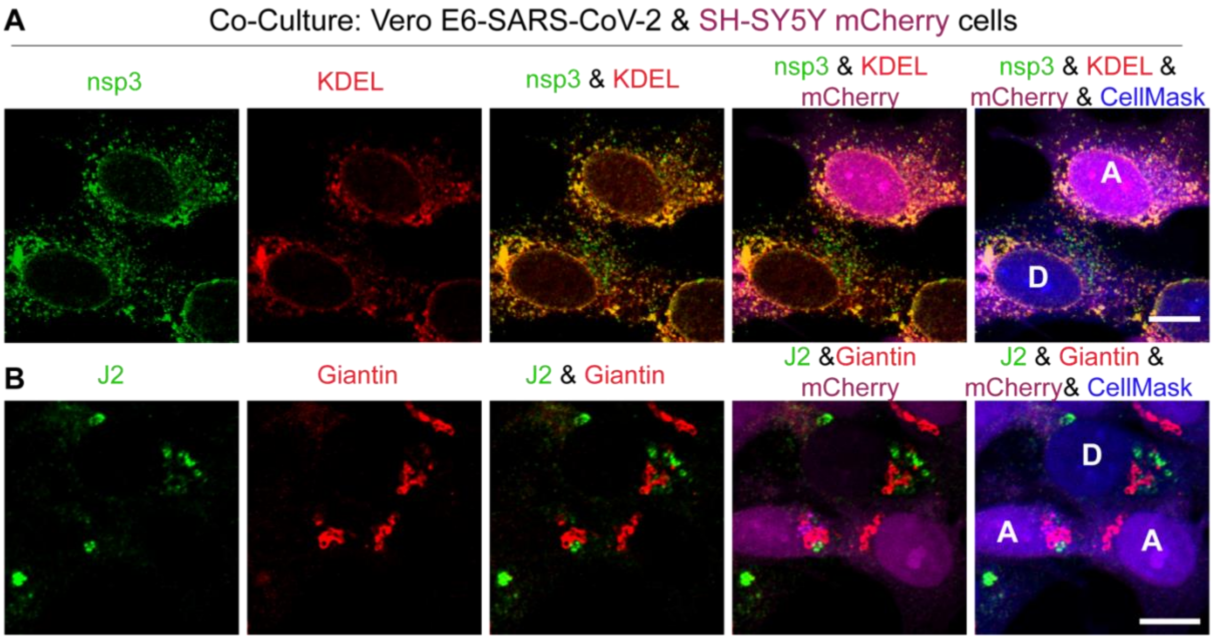

Fig. S4. Colocalization of anti-dsRNA (double-stranded RNA) J2 and the anti-non-structural protein 3 (nsp3) antibodies with ER/Golgi markers in SH-SY5Y cells co-cultured with SARS-CoV-2-infected Vero E6 cells. (**A-B**) SARS-CoV-2 infected Vero E6 cells (donor cells) were co-cultured for 48h with SH-SY5YmCherry acceptor cells. SH-SY5YmCherry are shown in magenta (**A**) Confocal micrographs showing staining with anti-nsp3 antibody used to detect DMV and an anti-KDEL antibody used to detect ER. (**B**) Confocal micrographs showing staining with J2 antibody used to detect dsRNA and Giantin antibody used to detect the Golgi. The letter A is for Acceptor and D is for Donor. Cell mask blue is used to delineate the cell body of each cell. Scale bars: 10µm.

Fig. S5

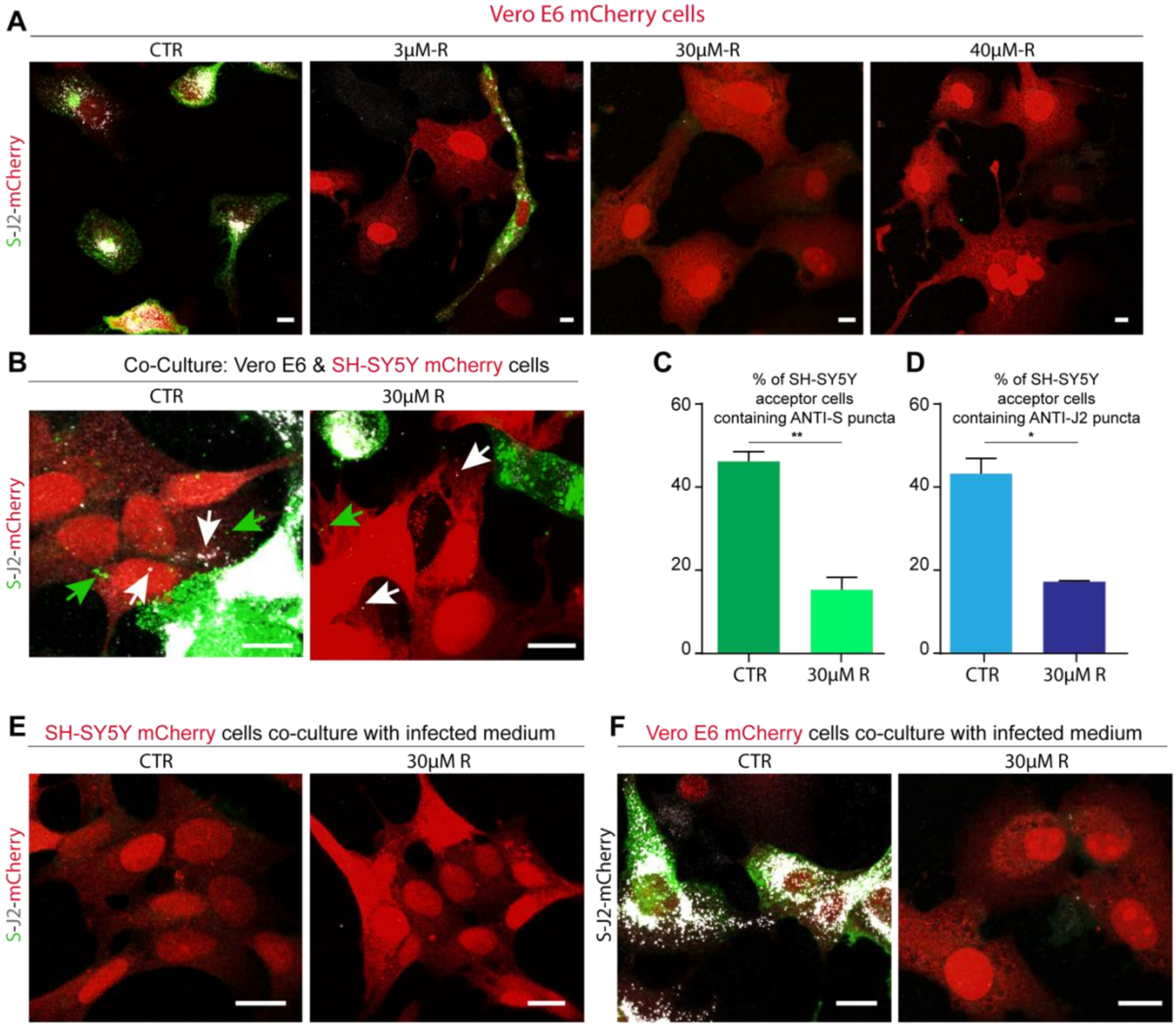

Fig. S5. SARS-CoV-2 can be transferred and newly synthesized in the acceptor cells. (A) Naïve Vero E6mCherry cells were pre-incubated for 1h at 37 °C with three different concentrations of Remdesivir (R) (3µM, 30µM, 40µM) prior to infection with 0.05 MOI SARS-CoV-2. The acceptors were in the presence of inhibitor throughout the time of infection. 48h post-infection, SARS-CoV-2 infected Vero E6mCherry cells were stained using J2 and anti-S to detect SARS-CoV-2 particles. **(B)** SARS-CoV-2 infected Vero E6 cells (donor cells) were co-cultured for 48h with SH-SY5YmCherry acceptor cells. SH-SY5YmCherry acceptor cells were pre-incubated with 30µM of Remdesivir (R) for 1h at 37 °C and 5% CO_2 before being co-cultured with infected Vero E6 cells. The co-cultures were maintained with the inhibitor and were fixed after 48h of incubation at 37 °C 5% CO_2 and immunostained with anti-S and J2 antibodies to detect SARS-CoV-2 particles. The white arrows indicate the J2 puncta detected in the cytoplasm of acceptor cells. The green arrows indicate the anti-S puncta detected in the cytoplasm of acceptor cells. **(C-D)** Graph showing the mean percentage of anti-S and J2 puncta

transferred in co-culture at 48h, treated or not with Remdesivir. **(E-F)** The supernatant from donor Vero E6 infected cells was added on top of (E) SH-SY5YmCherry (F) and Vero E6mCherry (previously incubated and/not with the inhibitor). After 48h of incubation, the secretion samples were fixed and immunostained for anti-S and J2 antibodies. Scale bars A: 20µm; B, E, F: 10µm.

Fig. S6

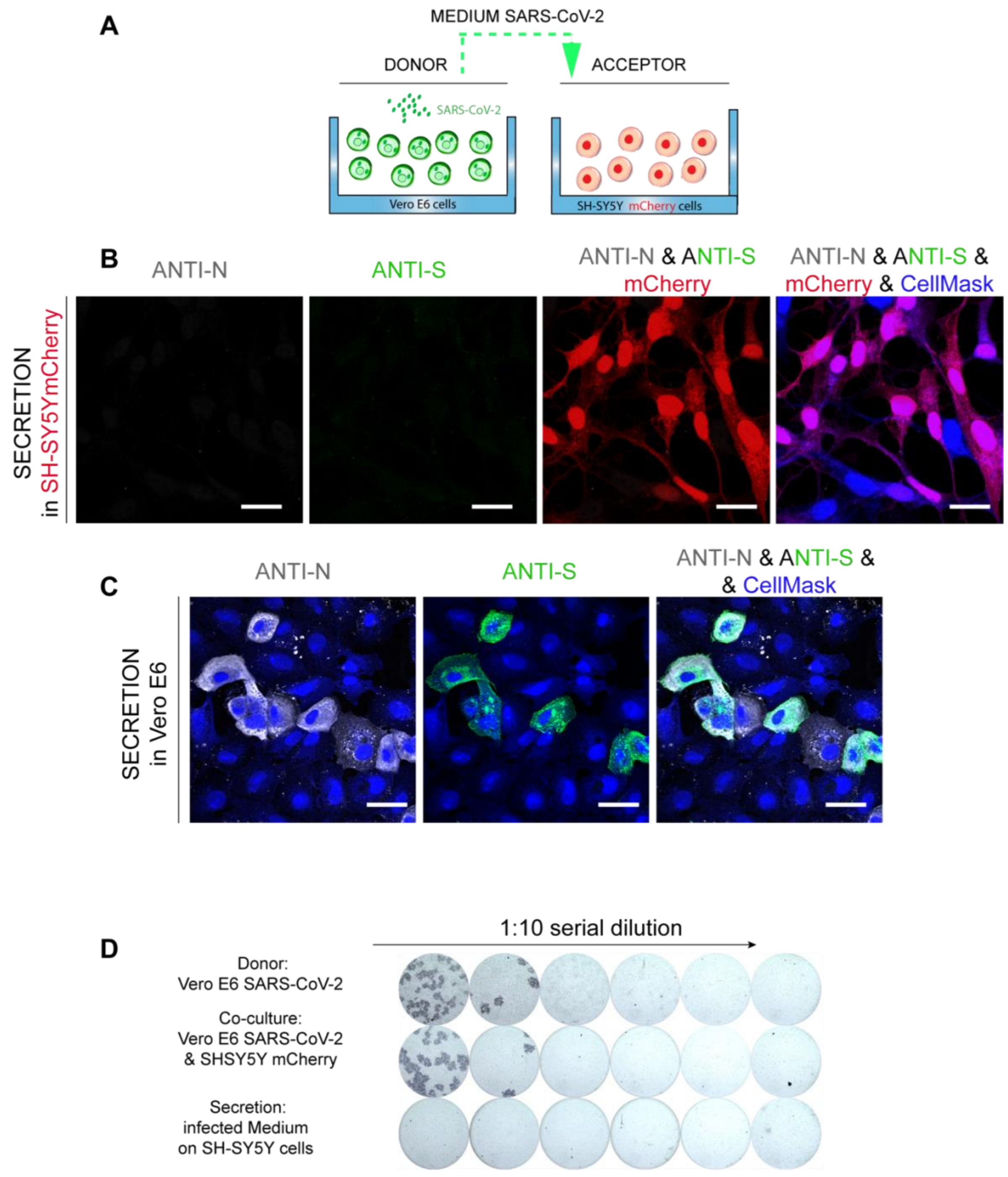

Fig. S6. Secretion test. (A) Description of secretion experiments: the medium from Vero E6 infected with SARS-CoV-2 MOI of 0.05 was centrifuged and incubated for 24h and 48h with non-permissive SH-SY5Y neuronal cells not infected Vero E6 cells (**C**). (**B)** Confocal micrograph showing SH-SY5Y cells incubated with infected medium from Vero E6 cells. Cells were fixed after 48h of incubation at 37° and 5% CO_2 and immunostained with anti-N antibody and anti-S antibody to detect SARS-CoV-2. (**C)** Confocal micrograph showing not-infected

Vero E6 cells incubated with infected medium from SARS-CoV-2 infected Vero E6 cells. Cells were fixed after 48h of incubation at 37° and 5% CO_2 and immunostained with anti-N antibody and anti-S antibody to detect SARS-CoV-2 particles. (**D)** The 48h supernatants from donor infected cells, from co-culture and from secretion test were used to assess viral production by focus forming assay titration protocol. Cell mask blue is used to delineate the cell body of each cell. Scale bars B, C: 20μm.

Fig. S7

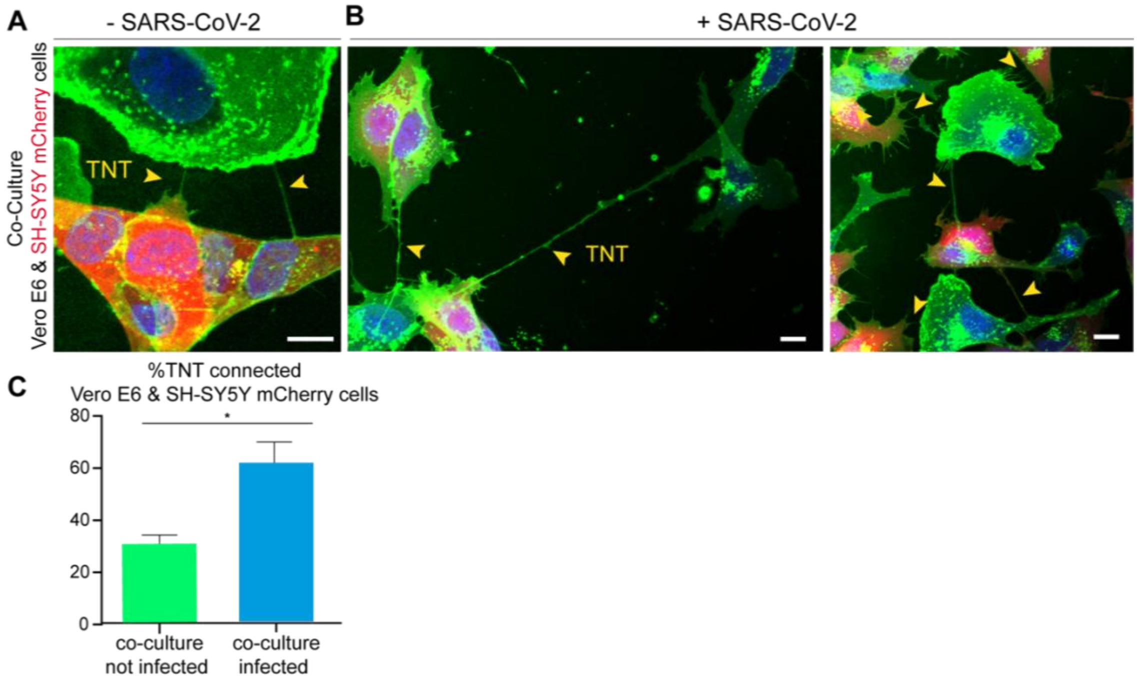

Fig. S7. SARS-CoV-2 infection increases the number of TNTs between infected Vero-E6 and SH-SY5Y cells. (A) Confocal micrograph showing TNTs between non-infected Vero E6 and SH-SY5YmCherry cells. (**B)** Confocal micrograph showing TNTs between SARS-CoV-2 Vero E6 infected cells and SH-SY5YmCherry cells. The yellow arrows indicate TNTs between Vero E6 cells and SH-SY5YmCherry cells. Cells were stained with WGA-488 to delineate the cell body of each cell and TNTs between cells. (**C)** Graph showing the percentage of TNT-connected cells between infected and/not Vero E6 cells and SH-SY5YmCherry cells. Mean percentage of TNT-connected Vero E6 non-infected cells and SH-SY5YmCherry cells: 35% ± 2.02 (N=3). Mean percentage of TNT-connected SARS-CoV-2-infected Vero E6 and SH-SY5YmCherry cells: 61.75% ± 6.27 (N=3) (*p=0.0154 for SARS-CoV-2-infected co-culture cells versus co-culture non-infected). Average length of TNTs of infected co-culture: 34.48μm SD=24.87; min=11.87μm, max=109.45μm). Average length of TNTs of control co-culture: 7.98μm SD=3.65). Scale bars: 10μm.

Fig. S8

A

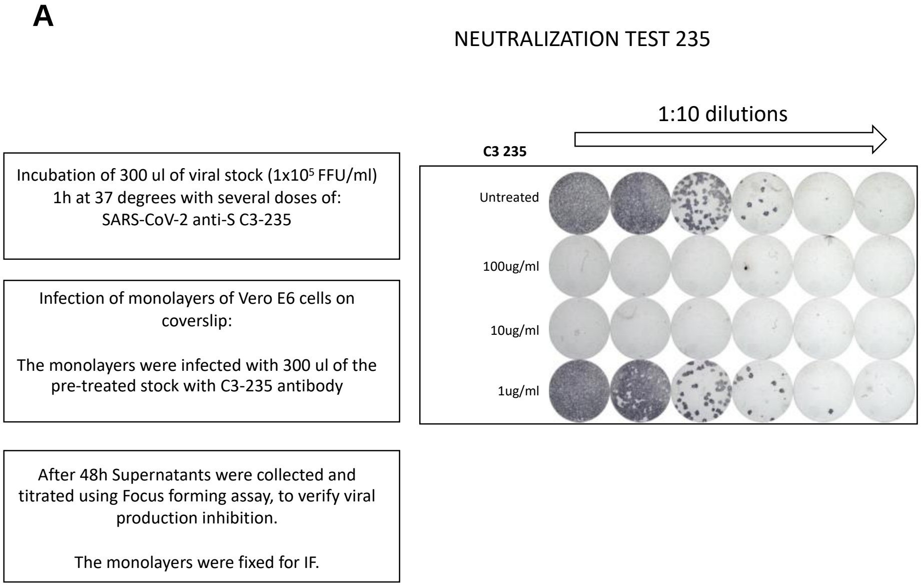

SAMPLE	FFU/ML
Neutr CTRL 1	9×10^5
100ug/ml 235	0
10ug/ml 235	0
1ug/ml 235	9×10^5

Fig. S8. Experimental set-up to identify the minimal concentration of SARS-CoV-2 IgG C3 235 sufficient to have a complete neutralization of the viral stock of 1-5 x 10^5 FFU/ml. (A) Three different concentrations of human SARS-CoV-2 IgG C3 235 (1, 10 and 100ug/ml) were incubated 1h at 37°C, 5% CO_2 and then used to infect monolayers of Vero E6 cells for 48h. Viral production was then assessed directly by processing the monolayers and titration of the supernatant by focus forming assay.

Fig. S9

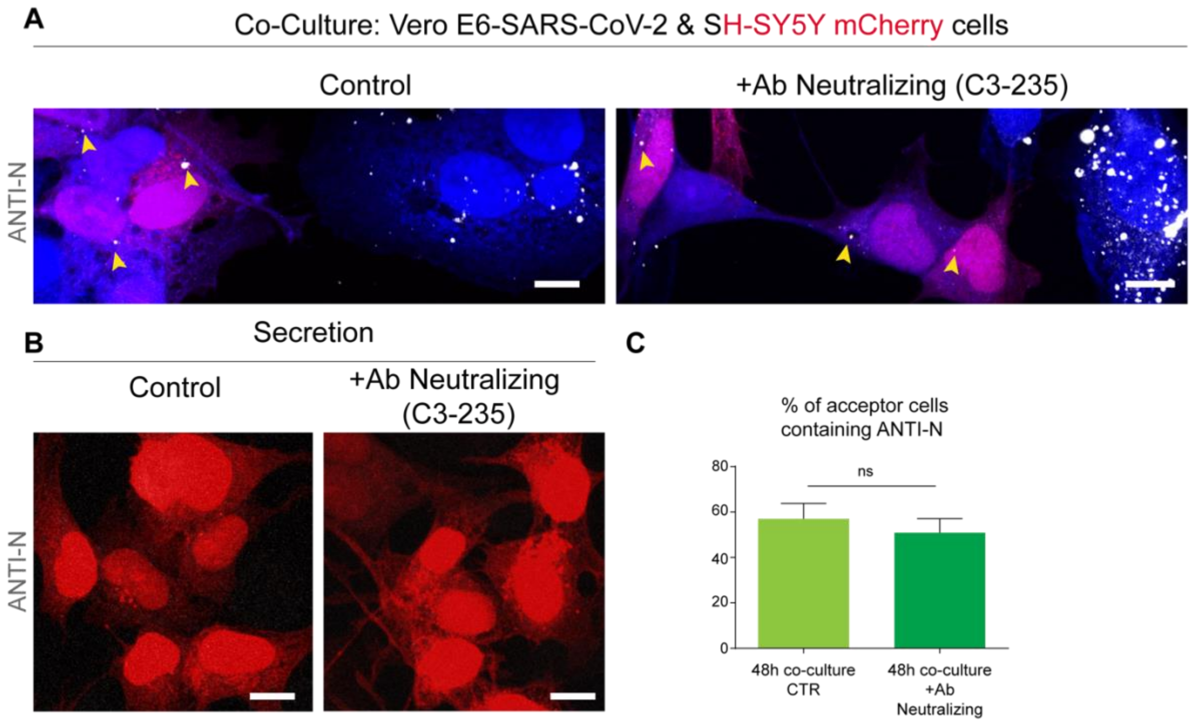

Fig. S9. SARS-CoV-2 viral particles spread between permissive cells to non-permissive cells through TNTs. (A) Vero E6 infected donor cells were co-cultured at 1:1 ratio with SH-SY5YmCherry acceptors in control conditions (without neutralizing antibody) and in neutralizing conditions. Donor Vero E6 infected cells were incubated with 10µg/ml of anti-SARS-CoV-2 IgG C3 235 for 1h at 37 °C 5% CO_2 before being co-cultured with Vero E6mCherry acceptors cells. The co-cultures were fixed after 48h of incubation at 37 °C 5% CO_2 and immunostained with anti-N antibody to detect SARS-CoV-2. Cellular cytoplasm was labelled with cell mask blue. The yellow arrowheads indicate the anti-N puncta detected in the cytoplasm of acceptor cells. (**B**) Secretion test; SH-SY5YmCherry cells were incubated with the supernatant deriving from donor Vero E6 infected cells. The supernatant from donor Vero E6 infected cells was incubated with 10µg/ml of the anti-SARS-CoV-2 IgG C3 235 for 1h at 37 °C, in order to neutralize the viral particles, before being added on top of SH-SY5YmCherry acceptor cells. After 48h of incubation at 37 °C 5% CO_2, the secretion samples were fixed and immunostained for anti-N. (**C**) Graph showing the mean percentage of anti-N puncta transferred in co-culture at 48h, treated and not with the neutralizing antibody. 48h co-culture control: 57.01% ± 3.95 (N=3) versus 48h co-culture plus neutralizing antibody: 50.92% ± 3.55 (N=3) (ns, p=0.3154 48h co-culture plus neutralizing antibody versus 48h co-culture control). Scale bars A-B: 10µm.

Fig. S10

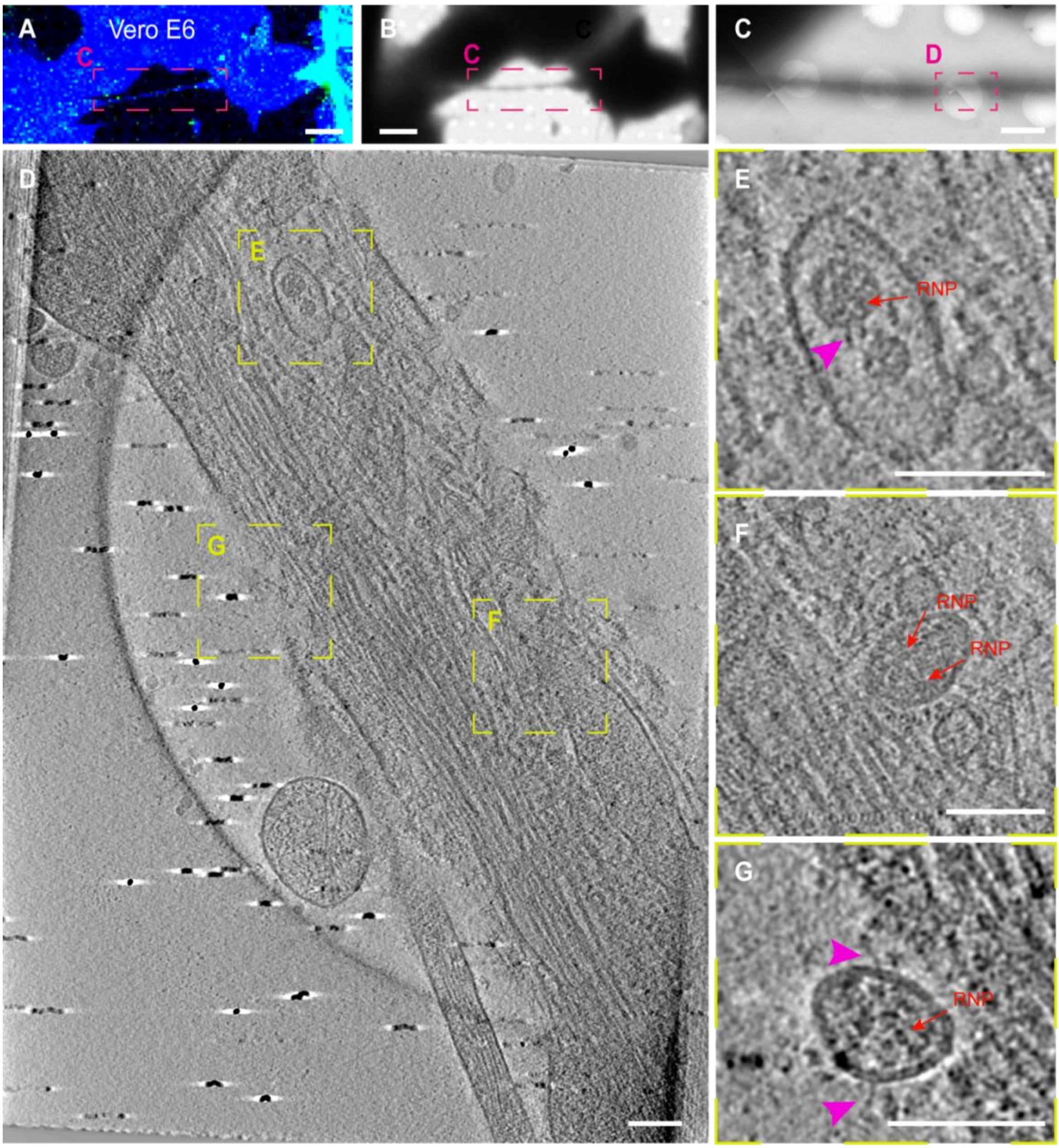

Fig. S10. Correlative IF (anti-S) cryo-EM strategies to discriminate SARS-CoV-2 localization in TNTs. (**A-G)** Cryo-EM grids were prepared using Vero E6 infected cells. (**A)** Confocal micrograph showing TNT connecting infected Vero E6 cells stained with anti-S antibody (green) and cell mask blue. Low (**B**) and intermediate (**C**) magnification TEM of (A). (**D)** High-magnification cryo-tomography slices showing vesicular compartments in correspondence of anti-S signal and SARS-CoV-2 virions inside and on TNT surface. (**E)** Enlargement of the high-magnification cryo-tomography slices (D) showing SARS-CoV-2-like structures inside a vesicle in the TNT lumen. (**F)** Enlargement of the high-magnification cryo-

tomography slices (D) showing SARS-CoV-2-like structure inside the lumen of a TNT. **(G)** Enlargement of the high-magnification cryo-tomography slices (D) showing SARS-COV-2-like structure on top of TNT membrane. RNP proteins and Spike are observed. Pink arrowheads indicate the spike; red arrows point the RNP proteins. Scale bars A, B: 15μm; C: 2μm, D-G: 100nm.

Fig. S11

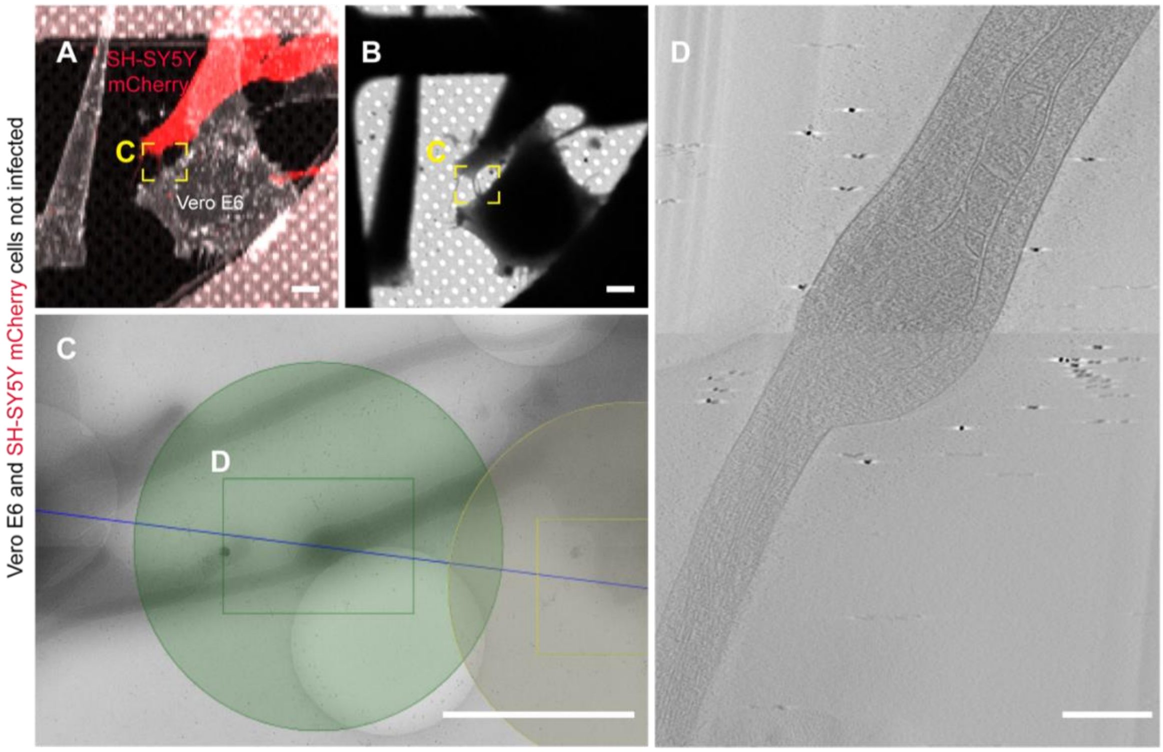

Fig. S11. Correlative Cryo-EM on TNT-connected not-infected Vero E6 and SH-SY5YmCherry cells. (A) Confocal micrograph shows TNT-connected naïve Vero E6 cells and SH-SY5YmCherry cells stained with WGA 633. **(B, C)** Low magnification (B) and intermediate magnification (C) of an electron micrograph of a TNT connecting naïve Vero E6 cells and SH-SY5YmCherry cells. **(D)** High magnification cryo-ET slice corresponding to the green rectangle in (C). Scale bars A, B: 10µm; C: 2µm; D: 100nm.

Fig. S12

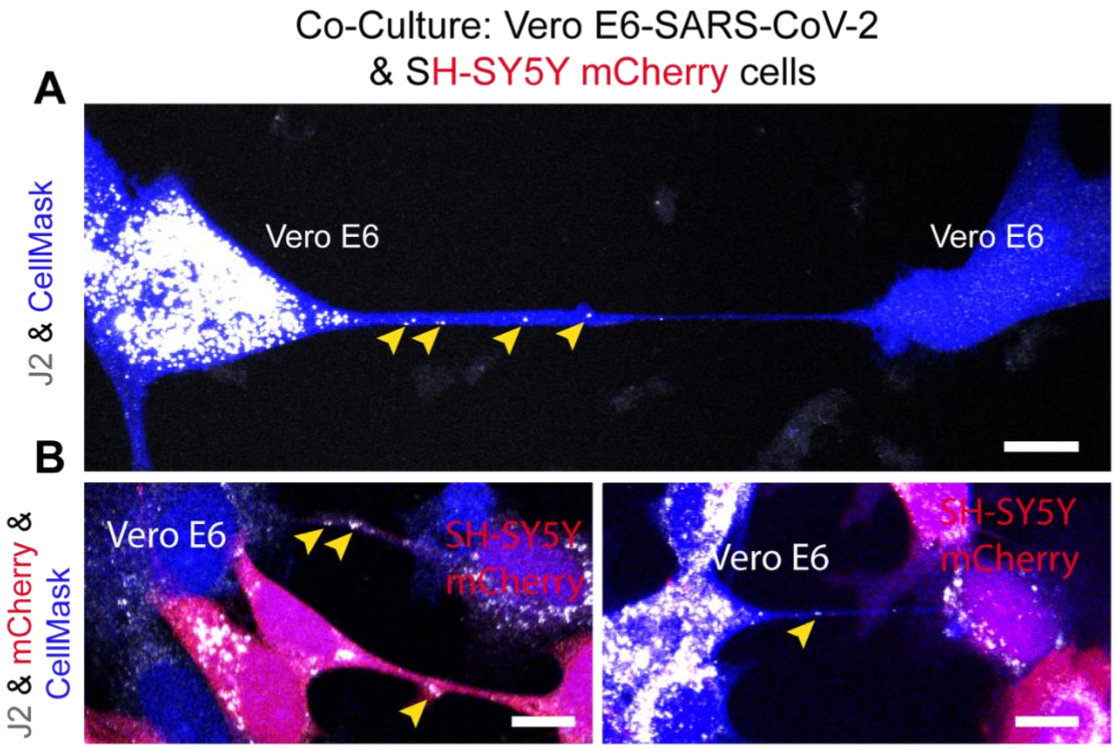

Fig. S12. Anti-dsRNA (double-stranded RNA) J2 antibody is detected in TNTs between SH-SY5Y cells co-cultured with SARS-CoV-2-infected Vero E6 cells. (A, B) SARS-CoV-2 infected Vero E6 cells (donor cells) were co-cultured with SH-SY5YmCherry cells (acceptor cells). Co-culture were fixed at 48h and stained with the J2 antibody to detect the virus. The yellow arrow points J2 particles in TNT between cells. **(A)** Confocal micrograph showing a TNT connecting Vero E6 infected cells. (**B**) Confocal micrograph showing a TNT connecting Vero E6 infected cells and SH-SY5YmCherry and TNTs between SH-SY5YmCherry. CMB is for cell mask blue that is used to delineate the cell body of each cells and TNTs between cells. Scale bars: 10µm.

Fig. S13

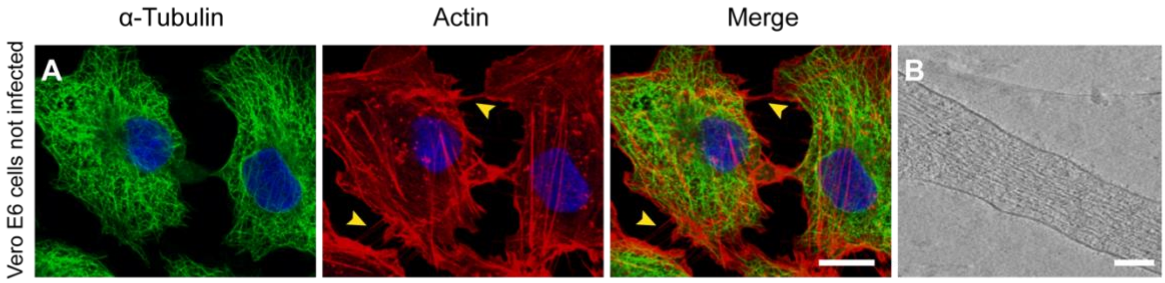

Fig. S13. Characterization of TNTs in Vero E6 cells by IF and cryo-EM. (**A**) Vero E6 cells were labelled with rhodamine phalloidin and anti-alpha-tubulin to detect actin filaments and microtubules, respectively. DAPI was used to label the nuclei. Arrowheads indicate TNTs. (**F**) Cryo-electron micrograph of TNT-connected naïve Vero E6 cells. Scale bars A, 10µm; B 100nm.

Description of Supplementary Movies

File Name: Movie S1

Description: Representative slices of a reconstructed tomogram displaying TNT connecting two SARS-CoV-2-infected Vero E6 cells shown in Fig. 6D. Scale bar: 200nm.

File Name: Movie S2

Description: Representative slices of a reconstructed tomogram displaying TNT arise from SARS-CoV-2-infected Vero E6 cells show in Fig. 6J. Scale bar: 200nm.

File Name: Movie S3

Description: Representative slices of a reconstructed tomogram displaying TNT arise from SARS-CoV-2 Vero E6 infected cells positive for the anti-Spike antibody containing vesicles compartments and SARS-CoV-2 like structure inside and on the surface shown in Fig. 7D. Scale bar: 200nm.

File Name: Movie S4

Description: Representative slices of a reconstructed tomogram displaying TNT arise from SARS-CoV-2 Vero E6 infected cells positive for the anti-Spike antibody containing vesicles compartments and SARS-CoV-2 like structure inside and on the surface shown in Fig. S10D. Scale bar: 100nm.

File Name: Movie S5

Description: Representative slices of a reconstructed tomogram displaying TNT between SARS-CoV-2 Vero E6 infected cells and SH-SY5YmCherry cells shown in Fig. 8D. These slices display Double Membrane Vesicles (DMV) inside TNTs. Scale bar: 200nm.

File Name: Movie S6

Description: Representative slices of a reconstructed tomogram displaying TNT between SARS-CoV-2 Vero E6 infected cells and SH-SY5YmCherry cells shown in Fig. 8I. These slices display several vesicular compartments inside TNTs. Scale bar: 100nm.

File Name: Movie S7

Description: Representative slices of a reconstructed tomogram displaying TNT between not-infected Vero E6 cells and SH-SY5YmCherry cells shown in Fig. S11D. These slices display mitochondria inside TNTs. Scale bar: 100nm.
